# Soluble β-Amyloid Oligomers Selectively Upregulate TRPC3 in Excitatory Neurons via Calcineurin-Coupled NFAT

**DOI:** 10.3390/cells14110843

**Published:** 2025-06-04

**Authors:** Zhengjun Wang, Dongyi Ding, Jiaxing Wang, Ling Chen, Qingming Dong, Moumita Khamrai, Yuyang Zhou, Akihiro Ishii, Kazuko Sakata, Wei Li, Jianyang Du, Thirumalini Vaithianathan, Fu-Ming Zhou, Francesca-Fang Liao

**Affiliations:** 1 Department of Pharmacology, Addiction Science and Toxicology, University of Tennessee Health Science Center, Memphis, TN 38163, USA; zwang138@uthsc.edu (Z.W.); dding3@uthsc.edu (D.D.); qdong4@uthsc.edu (Q.D.); mkhamrai@uthsc.edu (M.K.); yzhou78@uthsc.edu (Y.Z.); ksakata@uthsc.edu (K.S.); tvaithia@uthsc.edu (T.V.); fzhou3@uthsc.edu (F.-M.Z.); 2 Department of Pharmaceutical Sciences, College of Pharmacy, University of Tennessee Health Science Center, Memphis, TN 38163, USA; jw4742@cumc.columbia.edu (J.W.); wli@uthsc.edu (W.L.); 3 Department of Cell Biology and Genetics, The School of Basic Medical Sciences, Fujian Medical University, Fuzhou 350001, China; 4 Department of Neuroscience, UConn Health, Farmington, CT 06030, USA; ishii@uchc.edu; 5 Department of Anatomy and Neurobiology, University of Tennessee Health Science Center, Memphis, TN 38163, USA; jdu15@uthsc.edu; 6 Department of Ophthalmology, Hamilton Eye Institute, University of Tennessee Health Science Center, Memphis, TN 38163, USA

**Keywords:** TRPC3, TRPC6, NFAT, soluble β-amyloid oligomers, excitatory neurons, neuronal hyperexcitation, Ca^2+^ overload, synaptotoxicity, Alzheimer’s disease

## Abstract

To investigate how dysregulated transient receptor potential canonical channels (TRPCs) are associated with Alzheimer’s disease (AD), we challenged primary neurons with amyloid-β (Aβ). Both the naturally secreted or synthetic Aβ oligomers (AβOs) induced long-lasting increased TRPC3 and downregulated the TRPC6 expression in mature excitatory neurons (CaMKIIα-high) via a Ca^2+^-dependent calcineurin-coupled NFAT transcriptionally and calpain-mediated protein degradation, respectively. The TRPC3 expression was also found to be upregulated in pyramidal neurons of human AD brains. The selective downregulation of the *Trpc6* gene induced synaptotoxicity, while no significant effect was observed from the Trpc3-targeting siRNA, suggesting potentially differential roles of TRPC3 and 6 in modulating the synaptic morphology and functions. Electrophysiological recordings of mouse hippocampal slices overexpressing TRPC3 revealed increased neuronal hyperactivity upon the TRPC3 channel activation by its agonist. Furthermore, the AβO-mediated synaptotoxicity appeared to be positively correlated with the degrees of the induced dendritic Ca^2+^ flux in neurons, which was completely prevented by the co-treatment with two pyrazole-based TRPC3-selective antagonists Pyr3 or Pyr10. Taken together, our findings suggest that the aberrantly upregulated TRPC3 is another ion channel critically contributing to the process of AβO-induced Ca^2+^ overload, neuronal hyperexcitation, and synaptotoxicity, thus representing a potential therapeutic target of AD.

## 1. Introduction

Alzheimer’s disease (AD) is pathologically characterized by extracellular amyloid plaques and cytoplasmic tau tangles, which are believed to contribute to synaptic loss, neuronal death, and ultimately cognitive impairment [1,2,3]. The amyloid hypothesis was originally proposed largely based on the compelling genetic evidence: familial mutations in genes encoding for amyloid precursor protein (APP) or presenilin are associated with the early onset of AD in humans, resulting in increased Aβ production and amyloid plaque pathology [4]. By now, paramount evidence supports soluble Aβ oligomers (AβOs), instead of the insoluble amyloid β fibrils which constitute the extracellular plaques, as an early/earliest trigger of synaptic damage and cognitive impairment in AD [5,6].

Over the past two decades, the prefibrillar diffusible soluble forms of AβOs are found to be more toxic than their insoluble fibrillar counterpart to synapses [7,8,9,10,11]. Soluble AβOs are found in the cerebrospinal fluid of AD patients [12]; the soluble AβO content of the human brain is better correlated with the severity of the disease than are the classical amyloid plaques containing insoluble Aβ deposits and fibril-free oligomers [13,14,15]. Notably, soluble AβOs begin to accumulate in the human brain one or two decades before any clinical symptoms of AD appear, correlating with synaptic loss [6,7,8,9,10,11]. The exposure of hippocampal neurons to synthetic AβOs [16] or to cell-derived AβOs [17] induces progressive synaptic loss. The soluble AβOs extracted directly from AD brains also inhibit long-term potentiation (LTP), enhance long-term depression (LTD), and reduce dendritic spine numbers when injected into rodent brains [18]. Furthermore, AβOs have been reported to induce marked neuronal loss and disrupt hippocampus-dependent memory in mice [19]. The exact mechanisms underlying how AβOs lead to neuronal dysfunction remain only partially understood.

Dysregulated calcium signaling occurs in the early stages of AD [20]. Our understanding of the mechanisms underlying the disrupted Ca^2+^ homeostasis remains incomplete. Soluble AβOs are reportedly shown to induce intracellular Ca^2+^ overload and trigger subsequent synaptic dysfunction, mitochondrial failure, oxidative stress, and ultimately neurodegeneration [21,22]. Besides the well-established roles of the NMDA receptors (NMDARs) in mediating AβO-induced Ca^2+^ overload in neurons [23], the potential involvement of the non-selective calcium-permeable transient receptor potential canonical [TRPC] channels in AD is understudied. The TRPC family consists of seven Ca^2+^-permeable non-selective cationic membrane channels within the TRP superfamily [24,25]. TRPC2 is a pseudogene in humans, and the remainder are subdivided into two subfamilies, TRPC1/4/5 and TRPC3/6/7, which are largely expressed in the central nervous system (CNS), especially in the developing cerebellum and hippocampus [26,27]. Despite the increasingly recognized roles of the TRPC family members in multiple age-related diseases [28,29], their roles in chronic neurodegeneration, such as AD, are relatively understudied [30,31]. Based on the notions that the activation of these channels (e.g., TRPC3/6/7 in particular) often results in the depolarization of the cell membrane and calcium influx [32,33], we speculate that dysregulated TRPCs contribute to the AD pathogenesis.

To start probing into this question, we utilized both cell-derived naturally secreted soluble AβOs as well as synthetic Aβ oligomers to challenge primary cultured neurons to determine expressional changes in the TRPC family members. Among the family, AβOs were found to differentially regulate TRPC3 from the other members. We therefore focused on investigating distinct roles of TRPC3 in mediating excitotoxicity and synaptotoxicity.

## 2. Materials and Methods

### 2.1. Chemicals

The following chemical compounds were purchased from Sigma-Aldrich, St. Louis, MI, USA: MK 801 (M107), Memantine (M9292), Ifenprodil (I2892), Nifedipine (N7634), Verapamil (BP720), CNQX (C239), ML218 (SML0385), FK506 (F4679), Cyclosporine A (32425), MG-132 (M7449), APB (D9754), and Carbachol (212385). The following were also acquired: APV (No. 0105, Tocris, Bristol, UK), U73122 (No. S8011, Selleckchen, Cologne, Germany), Brefeldin A/BFA (B7450, Invitrogen, Waltham, MA, USA), Pyr3 (HY108465, MedChemExpress, Monmouth Junction, NJ, USA), and Pyr10 (Hy19408, MedChemExpress). 

### 2.2. Human Brain Specimens for Western Blots, RT-qPCR, and Immunohistochemistry

For Western blot analysis and reverse transcription–quantitative polymerase chain reaction (RT-qPCR), we used a cohort of human specimens provided from the Human Brain and Spinal Fluid Resource Center in year 2010 which is sponsored by NIHDS/NIMH, the National Multiple Sclerosis Society, and the Department of Veterans Affairs. The RNA from these human brains was extracted in 2010 shortly upon receiving the PFC tissues using the RNeasy Kit (Qiagen, Valencia, CA, USA) with on-column DNAase and was converted to cDNA using the Transcriptor First Strand cDNA Synthesis Kit (Roche, Indianapolis, IN, USA). We selectively used the 6 NC and 7 AD brain PFC tissue (Table S1) with an RNA integrity number (RIN) of 7 or higher for the recently performed RT-qPCR, with the average age being 69.0 ± 14.9 for AD and 76.6 ± 9.6 for the controls. From the same large cohort of patient specimens, we chose 16 human brain hippocampal tissue blocks (Table S2) to prepare protein lysates for Western blot analysis, consisting of 9 AD and 7 NC with the average age of individuals being 68.7 ± 20.9 and 79.3 ± 10.3, respectively. For immunohistochemistry, we used a separate cohort of specimens. A total of 9 human brain prefrontal cortical (PFC) tissue blocks, collected from short-postmortem interval (<6 h PMI) autopsies from the University of Kentucky AD Center/UK-ADC cohort, were used for immunostaining. Tissue specimens consist of 5 AD (with mixed vascular pathology) and 4 non-AD controls (NC), with the average age of individuals with AD being 85.3 ± 5.6 and NC being 89.2 ± 8.3 (Table S3). These tissue blocks were preserved in formalin and processed for paraffin embedding. Serial sections of 6 μm thickness were used for staining with anti-Aβ antibody (1:1000, 4G8, Sigma) and anti-TRPC3 antibody (1:1000, Alomone, Jerusalem, Israel, ACC-016) after using Citrate Buffer for antigen retrieval (Sigma, C9999), followed by counter-stain with Hematoxylin, developed using HRP/DAB IHC Detection Kit (Abcam, Cambridge, UK, ab64261).

### 2.3. Primary Hippocampal Cell Cultures

Neuronal cultures. Primary hippocampal and cortical neurons were isolated from E17 embryos of Sprague Dawley rats as described [34,35,36]. Briefly, the hippocampi and cortices were dissected and treated with trypsin and DNase by immersion in HBSS dissection buffer with 5 mL of 0.25% Trypsin–EDTA supplemented with 150 μL of DNase. The samples were incubated for 15 min at 37 °C, washed with HBSS dissection buffer, and spun down in 200 g for 2 min at room temperature (RT). The resulting cells were maintained in a Neurobasal medium (Thermo Fisher Scientific, 21103049) supplemented with 0.8 mM/L-glutamine and B27. All experiments presented in this work were performed on mature neuronal cells at 14 days in culture (14 DIV), unless specified otherwise (e.g., Figure S1B): Hippocampal neurons were used in immunocytochemistry and calcium imaging in 24-well plates (2 × 10^4^ cells per well) on Poly-D-lysine-coated (A3890401, Sigma) coverslips, and cortical neurons were used for RT-qPCR (1 × 10^6^ cells per well in 6-well plate), Western blot analyses (5 × 10^6^ cells per 10 cm dish), and Chromatin immunoprecipitation/ChiP analysis (1–2 × 10^7^ cells per 15 cm dish), respectively. Glial cell cultures. For rat primary glial cells, the dissociated cortical and hippocampal cells were resuspended in DMEM medium supplemented with 10% FBS and 2 mg/mL glutamine then plated in 75 cm^2^ flasks pre-coated with Poly-D-lysine. After reaching 80–90% confluency, the mixed glial culture was subjected to a shaking step at 37 °C at 200 rpm for 2 h to detach microglial cells. The supernatant containing microglia was collected, centrifuged at 1000 rpm for 10 min, and resuspended in fresh DMEM medium. The cells were then plated in 24-well plates at a density of 50,000 cells per well for immunocytochemistry. The remaining adherent astrocytes were digested with trypsin, centrifuged at 1000 rpm for 5 min, and resuspended in DMEM. The purity of the cultured cells was confirmed by immunostaining with antibodies against Neu N (NBP192693, Novus Biologicals, Toronto, ON, Canada), GFAP (G3893, Sigma), and IBA1 (01919741, Fujifilm WaKo, Osaka, Japan). We used anti-IBA1 (ab289874, Abcam) in co-staining with anti-TRPC3 antibody (ACC-016, Alomone). All experiments and analyses described below were performed in duplication on 3–6 batches of cultures.

### 2.4. Naturally Secreted AβO-Containing Conditioned Medium (7PA2 CM), Immunodepletion, and Synthetic Aβ Oligomerization

The 7PA2 cell line represents Chinese Hamster Ovary (CHO) cells stably transfected with human APP751 which contains a Val717Phe Mutation [37]. Conditioned medium (CM) containing naturally secreted AβOs was collected from 7PA2 and parental CHO cells as previously described [37]. 7PA2 CM and CHO CM were prepared by culturing 7PA2 or CHO cells. The culturing medium is DMEM (Thermo Fisher Scientific, 11965092), containing 10% FBS (Cytiva HyClone, Wilmington, DE, USA, SH30071.03IR25), 200 µg/mL G418 (Gibco, Waltham, MA, USA, 10131035), and 1% Pen/Strep (Sigma-Aldrich, P4333). Upon 80% confluency, Neurobasal medium was used to collect CM after 24 h incubation, and the collected medium was filtered (Millex, Duluth, GA, USA, SLG004SL) and stored at −80 °C until use. Immunodepletion of Aβ species using two antibodies against N-terminal and mid-region by BAM-10 (Sigma, AA1-10) and 4G8 (Sigma, AA17-24) was performed by repeated immunoprecipitations as described [34]. The lyophilized Aβ_25–35_ peptide (AnaSpec, Fremont, CA, USA) was dissolved in sterilized water (pH 7.4) to a final concentration of 250 µM. Soluble oligomeric and fibrillary Aβ_25–35_ was prepared by incubating the Aβ solution at 4 °C for 24 h. The fibrillar Aβ_25–35_ was prepared by incubating at 37 °C for 24 h and then spinning at 14,000 g for 10 min to sediment the insoluble fibrils [34]. The protein concentration in the supernatant was determined by a BCA protein assay kit (Thermo Fisher Scientific, Waltham, MA, USA) to confirm that over 90% of the Aβ_25–35_ peptides were fibrilized and precipitated. Fibrils were resuspended in water by vigorous vortexing prior to pipetting aliquots for cell stimulation.

7PA2 treatment. We used 1:10 diluted CM in cultured models for various lengths of time: typically 4 h for TRPC3/6 immunocytochemistry and overnight (16 h) for the determination of MAP2/ROS/apoptosis, unless it was indicated otherwise (i.e., in time course studies).

### 2.5. RNA Isolation and RT-qPCR

Total RNA was isolated using a Trizol reagent (Invitrogen, Carlsbad, CA, USA). Briefly, cells cultured on a 6-well plate were lysed in 1 mL of Trizol reagent. Samples were segregated into phenol–chloroform phases. The aqueous supernatant phase was transferred to an RNase-free tube and precipitated with isopropanol. The RNA pellet was washed with 70% ethanol prepared with DEPC-treated water, air dried, and dissolved in RNase-free water (Thermo Fisher Scientific, Waltham, MA, USA). The cDNAs were synthesized from the prepared total RNA using a High-Capacity cDNA Reverse Transcription Kit (4368814, Invitrogen) according to the manufacturer’s protocol. The amount of mRNA was detected with SYBR Green supermix (1725271, Bio-Rad, Hercules, CA, USA) and LightCycler Instrument (Roche). The relative amount of the TRPC family sample was calculated by the formula 2-ΔΔCt and normalized by the βActin (Table 1).

### 2.6. siRNA Transfection in Primary Neurons

Primary neurons were seeded in both 24-well and 6-well plates and transfected with siRNA3/6/scramble (20 µM stock/40 nM final concentration) mixing with Lipofectamine 3000 (Invitrogen) at ratios of 1:0.6 and 4:2.5 µL, respectively, at 5 DIV. After 6 h of incubation, the cells were replaced with fresh culture medium and harvested 7–9 days after transfection for MAP2 staining and for real-time RT-qPCR/Western bot analysis.

### 2.7. Fluorescent Immunocytochemistry

Primary neurons seeded on coverslips were fixed with 4% paraformaldehyde prepared in PBS at RT for 15 min. After brief washing, neurons were blocked and permeabilized in PBS containing 5% goat serum and 0.1% Triton X-100 at RT for 1 h in a 24-well plate. Anti-MAP2 (1:1000, 4403, Sigma), Anti-TRPC3 (1:1000, ACC-016, Alomone), TRPC6 (1:1000, ACC-017, Alomone), NFATc4 (1:1000, sc-271597, Santa Cruz, Santa Cruz, CA, USA), CaMKIIα (1:1000, ab22609, Abcam) antibody, or VGAT antibody (1:1000, Thermo Fisher, PA5-27569) was applied to the coverslips and incubated overnight at 4 °C. After extensive washes, Alexa 488 or 594-conjugated anti-rabbit or anti-mouse antibody (1:1000, Invitrogen) was applied and incubated for 1 h at RT. Slides were mounted with Fluoromount medium (Sigma, St Louis, MO, USA) prior to the image capturing under a high-resolution microscope (KEYENCE) or Olympus FV1000 confocal laser scanning biological microscope (Olympus Life Science, Tokyo, Japan).

### 2.8. The Quantification of Images Captured by the KEYENCE Microscope

**Figure 1:**
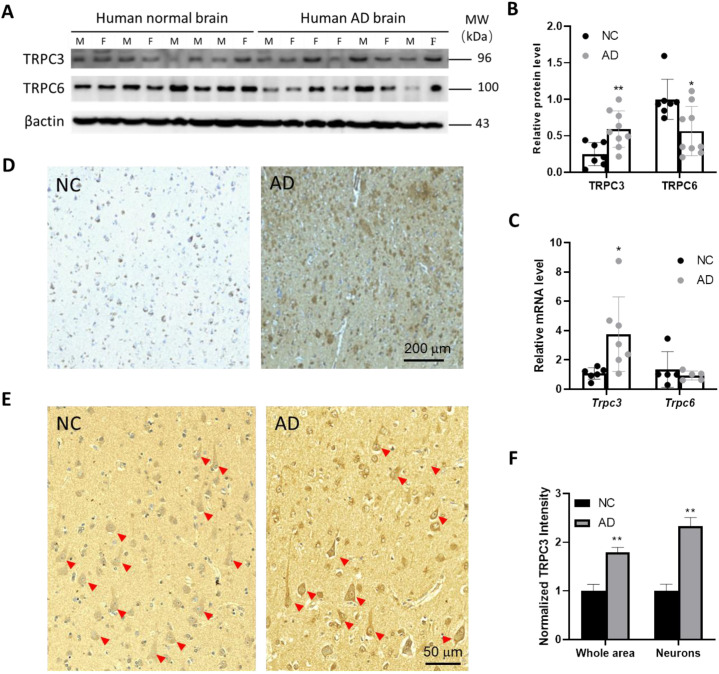
TRPC3 expression is upregulated in human brains. (**A**,**B**) Representative Western blots of TRPC3 and TRPC6 protein expression from 9 AD and 7 non-AD control (NC) hippocampal tissue samples. ** *p* < 0.01. F: female; M: male. (**C**) Relative mRNA levels of *Trpc3* and *Trpc6* genes were determined by RT-qPCR based on 7 AD and 6 NC samples of human AD brain PFC regions; only RIN > 7 samples were used. * *p* < 0.05. (**D**) Representative microscopic images displaying immunostained amyloid pathology (with 4G8 antibody). (**E**) Representative TRPC3-immunostained microscopic images of AD and control brains. Red arrowheads indicate typical cells with morphologies of large pyramidal neurons, respectively. (**F**) Quantification of TRPC3 DAB immunosignals based on 5 AD and 4 NC cases. ** *p* < 0.01. Note: human brain samples used for panels (**A**-**C**) were from same cohort of patient and control individuals, and representative images shown in panels (**D**,**E**) were taken from same pair of AD and NC cases from different cohort of clinical samples.

Human brain TRPC3 staining (Figure 1F). Initial images were taken from the immunostained brain sections (<1 cm × 1 cm in diameters), and two representative fields were chosen (image size: 1920 × 1440) from each of the frontal cortical areas to be used for determination of the relative TRPC3 immunosignal intensity in AD versus NC specimens (4 NCs and 5 AD). The captured images were analyzed using the ImageJ software (version 1.53e). A fixed intensity threshold was applied to define the DAB staining, after binary transformation to 8-bit black and white images. DAB signals were quantified by two approaches: 1) the percentage of the area covered by DAB staining was measured and compared between the different AD and NC samples and 2) the mean DAB signals were compared between the two groups from the selected pyramidal neurons (>80–100 neurons used from each group).

**Figure 2:**
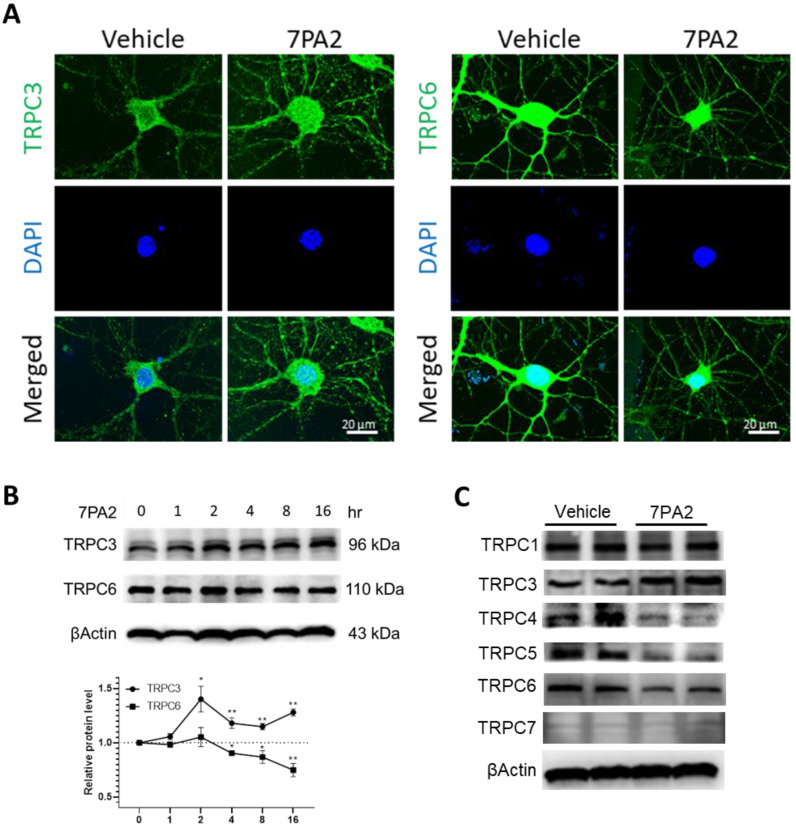
7PA2 treatment upregulates TRPC3 and downregulates TRPC6 expression in cultured primary neurons (14 DIV). (**A**) Representative TRPC3 and TRPC6 immunostaining images of 7PA2-treated neurons. (**B**) Representative Western blots and quantification based on 3 independent experiments. * *p* < 0.05; ** *p* < 0.01. (**C**) Representative TRPC family immunoblots of cellular lysates from cultured neurons at 4 h after 7PA2 treatment.

Data quantification in Figure S2. Dihydroethidium (DHE)-positive ROS levels. Quantification was based on the mean fluorescent signals of the original images taken (KEYEKE 10× magnification), using two representative fields from each experimental sample (triplicated from each batch of neurons). Annexin V-stained apoptotic cells. Quantification was based on the annexin V staining performed on live cells, and thus both the fluorescent and the light images were captured from the same corresponding field (two representative images at 20× magnification from each experimental well of 24-well plates). We counted the number of the green fluorescent positive cells over a total of 120–150 live cells as the percentage of apoptotic cells. Dendritic density and length of MAP2-stained neurons. Dendritic density was determined by manual counting of the number of dendritic branches and plotted average from >20 neurons from each group. We used NIH ImageJ to determine the length of the representative dendrites from each neuron (2–3 dendrites per neuron, <2–3 μm distal from soma), which provides a relative measure of each neuron’s dendritic branching and average length. We chose not to average the length of all the dendrites from each neuron because for some dendrites it is difficult to see which neuron they are derived from, as shown in Figure S1D. We believe that quantification based on these average dendritic lengths from a total of 25–30 neurons per treatment group can accurately reflect the overall dendritic complexity altered by 7PA2 treatment, pertinent to Figure S1C, Figure 2 and Figure 7A–D.

**Figure 3:**
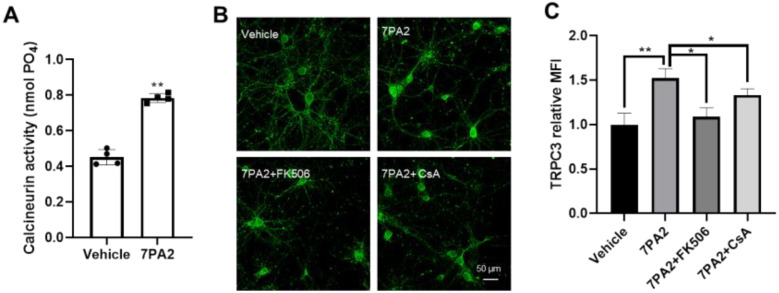
7PA2-induced TRPC3 upregulation is mediated by overactivated calcineurin (CaN). (**A**) Calcineurin’s phosphatase activity was induced by 2-fold, as determined 1 h after 7PA2 treatment. (n = 4). ** *p* < 0.01. (**B**) Representative TRPC3 immunocytochemistry showing preventive effects of two CaN inhibitors against 7PA2-upregulated TRPC3 expression (4 h time point). FK506 and CsA were used at 12.5 nM and 1 μM, respectively, in co-treatment with 7PA2. (**C**) Quantification of experiments of panel B (N = 3 independent experiments). * *p* < 0.05 and ** *p* < 0.01, respectively.

Immunosignals from the TRPC1-7 family members (Figure S3B,C). To quantify altered immunosignals from the TRPCs in the 7PA2-treated cultured cells (Figure S3B), we took microscopic images (20× magnification) from duplicated experiments and used two fields from each image and plotted the mean fluorescence intensity (MFI) of the TRPC levels (green) normalized against total cells (DAPI blue). To further quantify altered TRPC expression in excitatory neurons (Figure S3C), we compared MFI of the green fluorescence from at least 20 selected neurons (with large size) from each group. Similarly, the altered TRPC3 expressional levels in neurons after co-treatment of 7PA2 with the two CaN inhibitors were quantified for Figure 3B in Figure 3C.

NFAT–nucleus-translocated neurons (Figure S4). We manually counted at least 100 neurons (with large soma) from each experimental condition to determine the percentage of the cells with nucleus-translocated NFAT (e.g., overlapped NFAT–red immunofluorescence with blue DAPI). Although we occasionally observed NFAT nucleus translocation in smaller cells (e.g., glia), we excluded them from our counting.

### 2.9. Western Bslotting

For TRPC3 and TRPC6 detection in primary cultured neurons, cell lysate was collected from frozen cultures with RIPA lysis buffer. Total protein samples were separated by gel electrophoresis and transferred to 0.2 µm PVDF membranes using the Bolt SDS-PAGE system (Life Technologies, Carlsbad, CA, USA). Membranes were blocked in 2% BSA in TBS supplemented with 0.01% Tween-20 (TBST) for 1 h RT, followed by incubation overnight at 4 °C in primary antibody diluted in 5% bovine serum albumin/PBST. The primary antibodies used were as follows (all from Alomone except for TRPC6, at 1:1000): TRPC1 (ACC-010), TRPC3 (ACC-016), TRPC4 (ACC-018), TRPC5 (ACC-020), TRPC7 (ACC-066), and TRPC6 (SAB4300572, Sigma). Membranes were then washed three times with TBST and incubated in HRP-conjugated secondary antibodies (1:4000, Thermo Fisher) diluted in 2% BSA/PBST at RT for 1 h. After washing, membranes were developed using SuperSignal West Pico Chemiluminescent ECL substrate (Thermo Fisher).

### 2.10. Calcineurin Enzymatic Activity Assay

Calcineurin activity was determined using a calcineurin cellular activity assay kit (Enzo Lifesciences, Farmingdale, NY, USA, BML-AK816-0001) following the manufacturer’s manual.

### 2.11. ChIP-PCR

To examine NFATc4 binding to Trpc3 and Trpc6 gene promoters, ChIP-PCR was conducted with or without 7PA2 treatment for 4 h. ChIP was performed using Chromatin Immunoprecipitation Assay Kit (cat#17–295, Millipore, Burlington, MA, USA), following the manufacturer’s instruction. Briefly, vehicle and 7PA2-treated primary neurons (2 of 15 cm dishes each condition) were incubated with 1% formaldehyde in PBS for 10 min at 37 °C to cross-link nuclear proteins to the DNA. Fixation was quenched by adding glycine at a final concentration of 125 mM and by quickly washing three times with ice-cold PBS. Next, the cells were harvested and homogenized by pipetting in SDS lysis buffer (1% SDS, 10 mM EDTA, 50 mM Tris, pH 8.0) containing protease inhibitors (1 mM PMSF and 1 μg/mL aprotinin). The samples were placed on ice for 10 min and then sonicated on ice. This resulted in genomic DNA fragments ranging in size from 200 to 1000 bp. The lysates were centrifuged at 13000 rpm for 5 min at 4 °C to remove insoluble material. Ten percent of the supernatant was kept for input. The remaining supernatant was 10-fold diluted with ChIP dilution buffer (0.01% SDS, 1% Triton X-100, 1.2 mM EDTA, 16.7 mM Tris–HCl, pH 8.0, and 167 mM NaCl) containing protease inhibitors and then was used for immunoprecipitation with an antibody-detecting NFATc4 (sc-271597, Santa Cruz) or mouse IgG (I5381-1MG, Sigma) as a negative control and incubated overnight at 4 °C. Chromatin–antibody complexes were collected with protein A agarose beads (cat#16-157C, Millipore) and were washed sequentially with low-salt, high-salt, and LiCl buffers (Millipore) then TE buffer (10 mM Tris and 1 mM EDTA pH 8.0). Chromatin was eluted with freshly made 1% SDS/NAHCO3 buffer. Chromatin samples and input were incubated at 65 °C for 4 h in high-salt conditions with shaking to reverse cross-linking. DNA fragments were treated with proteinase K followed by extraction with phenol/chloroform/ethanol then subjected to PCR.

### 2.12. Single-Nucleus RNAseq Data Analysis

We performed the unbiased single-nucleus RNA sequencing (snRNAseq) using nuclei samples isolated from the cortex and hippocampi region of ~5-month-old female mice from App^NL-G-F/wt^ mice and littermate controls [38]. We were able to divide the nuclei into 37 clusters and annotated them using the marker genes, including excitatory neurons (Slc17a7), inhibitory neurons (Gad2), astrocytes (Aqp4, Clu), microglia (Cx3cr1, Hexb), OPC (Pdgfra), and OLs (Mog). The excitatory neurons and inhibitory neurons were subjected to differential gene expression analysis. The details of the downstream snRNAseq analysis for the DEG are described separately in Supplementary Materials file.

### 2.13. Electrophysiology

Overexpression of mouse *Trpc3* gene (GenBank: AK080619.1) in hippocampal CA1 of mouse brains. We injected AAV2/9-CAG-mTRPC3 (AAV-mTRPC3) (4.06E + 11 vg in 0.5 μL) and control AAV2-CAG-eGFP (AAV-eGFP) viruses to each side of five female C57BL/6 mouse brains at 2 months old, following the same surgical procedure of stereotaxic microinjection using KOPF microinjection unit (Model 5000) as described in our prior work [35,36], using coordinates AP= −2 mm; ML= +1.5 mm; and DV= −1.75 mm with respect to bregma, at speed of 0.15–0.2 μL/min with injection needle remaining for additional 3 min. Viral production was outsourced by the Viral Core of Iowa University.

Hippocampal brain slice preparation. The five virally injected mice (5 months old) were euthanized by decapitation under deep isoflurane anesthesia. The skull was quickly opened, and ice-cold cutting solution was poured onto the brain to cool the tissue. The brain was quickly dissected out and immediately immersed in an oxygenated ice-cold cutting solution containing the following (in mM): 220 glycerol, 2.5 KCl, 1.25 NaH2PO4, 25 NaHCO3, 0.5 CaCl2, 7 MgCl2, and 20 D-glucose [39]. Four hundred (400) μm-thick coronal brain slices were cut on a Leica Zero Z VT1200S vibratome (Leica Microsystems, Wetzlar, Germany). The brain slices (2–3) containing dorsal hippocampus were further bisected at the mid-line and transferred to a holding chamber filled with a standard artificial cerebrospinal fluid (aCSF) (in mM: 125 NaCl, 2.5 KCl, 25 NaHCO3, 1.25 NaH2PO4, 2.5 CaCl_2_, 1.3 MgCl_2_, and 10 D-glucose) that was kept at 34 °C and continuously bubbled with 95% O_2_ and 5% CO_2_ to supply oxygen and maintain pH at 7.4 After an initial 30 min incubation at 34 °C, the brain slices were kept at room temperature (22 °C). For recording, KCl in the aCSF was 4 mM and the temperature was 30 °C, maintained by an in-line solution heater. Ascorbic acid (vitamin C) (0.4 mM) was included in all brain slice bathing solutions to protect the tissue.

Extracellular recording. Slices were placed in a recording chamber mounted on the home-made stage of a fluorescence microscope (Olympus, Tokyo, Japan, BX51WI) and continuously perfused at 2 mL/min with 4 mM KCl-containing aCSF saturated with 95% O2 and 5% CO2. Recordings were made at 30 °C, maintained by a temperature controller (TC 324B, Warner Instruments, Hamden, CT, USA). Glass pipettes/electrodes were pulled from borosilicate glass capillary tubing (cat. # B150-110-10, Sutter Instrument, USA) using a PC-10 puller (Narishige, Tokyo, Japan) and had resistances of 2–3 MΩ when filled with aCSF (measured in voltage clamp before and after extracellular recording). A Multiclamp 700B amplifier, pClamp 9.2 software, and Digidata 1322A interface (Molecular Devices, Sunnyvale, CA, USA) were used to record electrical activity in the CA1 region of the brain slice, and the data were digitized at 5000 Hz and stored in the computer hard disk. Recordings were made in current clamp mode. The TRPC3 agonist GSK1702934A (GSK) (cat. # 6508, Tocris, Ellisville, MI, USA) and fast voltage-gated sodium channel blocker tetrodotoxin citrate (TTX) (cat. # 1069, Tocris) were each bath-applied. Off-line, the spontaneous neuronal spikes during baseline, GSK, and TTX (2 min segment for each condition) were detected by Clampfit (using the threshold detection method). The detected spikes were visually verified.

### 2.14. Calcium Imaging

The experimental setup for Figure S7E used Oregon Green 488 BAPTA-2/AM (Invitrogen, O6809) and the following recording conditions: Extracellular solution was prepared to contain (in mM) 140 NaCl, 6 KCl, 2 CaCl_2_, 1 MgCl_2_, 10 glucose, and 10 HEPES. Divalent-free solution was prepared to contain (in mM) 145 NaCl, 5 EGTA, 2 EDTA Na+ salt, 10 glucose, and 20 HEPES. Oregon Green 488 BAPTA-2/AM (Invitrogen, O6809) was diluted into the solutions at a working concentration of 4–5 µM. Cells were incubated with the above solutions for 1 h. Fluorescent imaging was performed using a Lambda 10-2 Optical Filter Changer Control System (Sutter Instrument, Novato, CA, USA) at a sampling rate of 500 ms, recorded by MetaFluor software (latest v. 7.7., Molecular Devices, San Jose, CA, USA).

### 2.15. Confocal Ca^2+^ Imaging (For Data Presented in Figure 7E)

Intracellular Ca^2^⁺ signals were quantified using Cal Red™ R525/650 (Cat#20590, AAT Bioquest, San Francisco, CA, USA), a ratiometric Ca^2^⁺ indicator with spectral properties similar to Fura Red. In the experimental setup for Figure 7E, Ca^2^⁺ measurements were acquired using a 30× silicon objective (NA 1.05) on an Olympus FV-3000 laser-scanning confocal system mounted on an Olympus IX-83 motorized inverted microscope (Olympus, Shinjuku, Tokyo, Japan) and controlled by Olympus FV31S-SW software (Version 2.6.1.243). To ensure consistency across experiments, acquisition parameters—including pinhole diameter, laser power, PMT gain, scan speed, optical zoom, offset, and step size—were held constant. Ratiometric Ca^2^⁺ signals were acquired using a Galvano scanner in non-sequential line scan mode with an excitation wavelength of 488 nm and emission detection at 500–540 nm and 650–750 nm. Imaging was performed at a line scan rate of 3.16 ms/line and a pixel dwell time of 8 μs, with a scan resolution of 256 × 256 pixels.

Cells exhibiting large Ca^2^⁺ responses to pharmacological treatments were classified as neurons, and regions of interest (ROIs) were placed on their soma. All analyses were performed using ImageJ (NIH, version 2.14.0/1.54f). Background fluorescence was subtracted, and the fluorescence amplitude (ΔF/F_0_) for each neuron was calculated as (F − F_0_)/F_0_, where F_0_ represents the baseline fluorescence. The fluorescent intensity of each neuron was determined as the average intensity within each ROI. The fluorescence ratio, R, was calculated as the ratio of F_520_ to F_647_ (fluorescence intensity at 488 nm excitation and two emissions at 500–540 nm and 650–750 nm); F520 signal and the ratio increase when intracellular Ca^2+^ concentration increases.

### 2.16. Statistics

Statistical analysis was performed using GraphPad Prism (Ver 8.3.0). Two-tailed *t*-tests were performed for comparisons between two groups, and one- or two-way ANOVAs were performed for more than two groups and multiple comparisons. All tests were conducted as two-sided tests with significance levels set as follows: ns, *p* > 0.05; *, *p* < 0.05; and **, *p* < 0.01.

## 3. Results

### 3.1. TRPC3 Expression Is Elevated in Post-Mortem Human AD Brains

We first determined the expressional profile of TRPC3 in post-mortem human AD specimens. As shown in Figure 1A–C, both the Western blots and RT-qPCRs analyses on frozen tissues from a cohort of AD and non-AD control (NC) cases (Tables S1 and S2) revealed more than a 2-fold increase in the steady-state levels of the TRPC3 protein and the *Trpc3* mRNA and reduced the TRPC6 protein but did not significantly alter the *Trpc*6 mRNA in AD samples. To investigate the cell-type specificity of the upregulated TRPC3 in AD brains, we then used immunohistochemistry to detect TRPC3 protein with diaminobenzidine (DAB) as the chromogen in human brain tissues. Indeed, the TRPC3 DAB immunosignals revealed significantly higher expressional levels of the TRPC3 protein in AD brain prefrontal cortical (PFC) specimens from a different cohort of patients compared to the non-AD controls (NCs) (Table S3, Figure 1D), most notably in those large pyramidal neurons (Figure 1E, red arrowheads). Notably, large pyramidal neurons represent the most abundant neuronal cell type in the prefrontal and cerebral cortex which use glutamate as their excitatory neurotransmitter.

Interestingly, the NC cases used in this immunohistochemical comparison were mostly associated with a medical history of vascular pathology, including multiple brain infarctions or vascular dementia. Notably, all five cases of AD used here display higher levels of the amyloid pathology in addition to a vascular pathology (Table S3, Figure 1D). The quantification of the increased TRPC3 expression in AD (Figure 1F) was based on the images collected from the brain PFC areas (N = 5 AD and N = 4 NC). We used two approaches to compare the relative DAB signals from the total brain cells within the chosen representative images versus the selected neurons featuring a pyramidal soma, as illustrated by the cells marked with the red arrowheads in Figure 1E.

### 3.2. AβOs Aberrantly Upregulate TRPC3 and Downregulate TRPC6 Expression in Mature Hippocampal Neurons

We recently reported the neuroprotective role of a pyrazole Pyr3-modified TRPC3 antagonist compound, JW65, against the AβO-induced dendritic loss of primary cultured hippocampal neurons [40]. This finding suggests a potential role of TRPC3 in soluble AβO-mediated synaptotoxicity. To better understand the possible role of TRPC3 in AD’s pathogenesis, we sought to investigate how AβOs may dysregulate TRPC3 expression in primary cultured models of mature rat hippocampal cells.

Using a primary culture model derived from dissected E17 rat embryos consisting primarily of hippocampal principal pyramidal excitatory neurons, we tested the treatment effects from a 7PA2 cell culture conditioned media (CM). The latter has been the best-characterized cellular model of naturally secreted AβOs to exert synaptic toxicity in multiple in vitro and in vivo assays [17,18,19,41,42,43,44], and the active components were largely reported to be primarily a mixture of lower n-AβOs in sub-nanomolar concentrations. We have successfully used the 7PA2 CM in our prior work in primary neuronal models for studying molecular and cellular mechanisms of delayed cell death (i.e., excitotoxicity) and synaptotoxicity (i.e., dendritic spine loss) and have identified several synaptoprotective mechanisms and chemical compounds [34,35,36]. A ten-fold diluted 7PA2 CM consistently resulted in neuronal morphological changes and cell death as we reported and thus was used throughout the subsequent experiments (referred to herein as the “7PA2 treatment”). As we reported [34], the mass spectrometry analysis revealed a mixture of Aβ species ranging from 37 to 42 amino acids in length, which displayed the typical profile of the lower n-AβOs, predominantly dimers, trimers, and tetramers, on Western blots (Figure S1A). The 7PA2 treatment only induced significant neurotoxicity in mature (14 DIV) and, at higher degrees, in aged neurons (21 DIV), while immature neurons (7 DIV) were resistant to this treatment, as determined by Annexin V (Figure S1B). Therefore, we used mature neurons in the subsequent experiments. Of note, the immunodepletion of 7PA2 CM with two anti-Aβ antibodies targeting both the N-terminus AA1-10 (BAM-10) and mid-AA17-24 (4G8) completely abolished its neurotoxicity as determined by multiple parameters, including reactive oxygen species (DHE), apoptosis (Annexin V), and dendritic loss (MAP2) (Figure S2) [34]. Together, these findings validated the AβO-mediated neurotoxic effects.

The microtubule-associated protein MAP2 has been established to be required during neuronal development (e.g., for neuronal process formation and for maintaining mature dendritic structures). It is preferentially localized to the dendritic arbor (but not the axon) and thus is commonly used as a marker [45,46] for detecting changes in the dendritic morphology and even functions [47]. In a time course study (Figure S1C), the 7PA2 treatment induced morphological changes in principle pyramidal neurons, with a noticeable dendritic loss or regression detected by fluorescent MAP2 staining at 8 h, as seen with discontinuous and thinning dendritic branches. We identified a critical duration between 16 and 24 h for the 7PA2 treatment to induce marked synaptic toxicity (i.e., >50–60% synaptic loss), which was determined by the dendritic length in particular. By 48 h, we detected soma shrinkage (i.e., reduced soma size) in the majority of the neurons. These morphological changes are predicted to be associated with their functional consequences on neuronal excitability and synaptic transmission, as documented for AβOs (e.g., regression and thinning) and spine loss, resulting in synaptic dysfunction [34,35,48]. All these parameters, including the soma size, dendritic length/density, and spine shape/density, are important correlates for synaptic strength and plasticity. However, dendritic length, especially when measured using MAP2 staining, is used more widely than the number/density for quantifying neuronal complexity because it provides a more comprehensive measure of dendritic arborization. It is thus a valuable indicator of synaptotoxicity in AD, which we chose as a major measurement in the following experiments. Figure S1D shows a typical microscopic image of the MAP2 staining in our neuronal culture, which contains more than 85–90% neuronal cells (NeuN-positive) and ~10% glial cells (GFAP-positive); less than 1% of the IBA1-positive cells survived to 14 DIV in the Neurobasal media (Figure S1E).

We then found that the 7PA2 treatment of cultured neurons rapidly induced the upregulation of the TRPC3 protein level, as detected by immunocytochemistry and confocal microscopy. A time course study revealed significantly upregulated TRPC3 after the 7PA2 treatment, which remained at high levels until at least 8 h and started declining to the basal levels at 16 h (Figure 2B). Interestingly, confocal imaging reveals that the 7PA2 treatment induces an upregulated TRPC3 expression in primary hippocampal neurons not only on the plasma membrane but also in dendritic processes. On the contrary, the 7PA2 treatment resulted in a reduced expressional level of TRPC6, a closely related TRPC member of TRPC3 (Figure 2A). This reciprocal effect between TRPC3 and TRPC6 was also confirmed by the Western blot analysis (Figure 2B); the latter also displayed a reduced expression of TRPC4/5 (Figure 2C), in addition to TRPC6 by AβOs. These changes were confirmed by immunocytochemistry, indicating that soluble AβOs induce differential regulation on the TRPC family members (Figure S3A–C).

We also performed the RT-qPCR to determine the mRNA expressional changes in the TRPC family genes upon the 7PA2 treatment in a time course study (Figure S3D). Notably, the *Trpc3* gene expression displayed a typical bell-shaped pattern, showing rapid upregulation starting from 0.5 h and peaking by 2 h after the 7PA2 treatment. This is consistent with the protein levels presented in Figure 2. The *Trpc6* gene showed a slight decline but did not reach significance. The RT-qPCR results also revealed expressional changes in the genes encoding for other TRPC members (i.e., *Trpc1*, *Trpc4*, *Trpc5,* and *Trpc7*), showing inconsistent results from their protein levels detected by Western blots (Figure 2C), which requires a further investigation of their dysregulatory mechanisms by AβOs at post-transcriptional and translational/post-translational levels.

### 3.3. AβOs Transcriptionally Upregulate Trpc3 Gene Expression via Ca^2+^-Dependent Calcineurin–NFAT Mechanism

We then searched for the reported mechanisms for the upregulated *Trpc3* gene under pathological conditions and found that both the TRPC3 and TRPC6 genes were reportedly upregulated under cardiac hypertrophy, a condition where the heart muscle thickens and stiffens the heart tissue [49] by the Ca^2+^/calmodulin-dependent phosphatase 2B (calcineurin/CaN) coupled nuclear factor of the activated T cells (NFAT) pathway. The closely related TRPC3 and TRPC6 subfamily members are non-selective calcium channels that are activated by diacylglycerol (DAG), a product of high phospholipase C (PLC) activity [50]. The activation of TRPC3 and TRPC6 induced by the angiotensin II-mediated PLC pathway leads to an influx of Ca^2+^ ions into the myocardial cells, which in turn activates the Ca^2+^-dependent CaN to dephosphorylate the NFAT, allowing it to translocate to the nucleus and activate the transcription of genes involved in cardiac hypertrophy, including TRPC3 and TRPC6. This creates a positive feedback loop, where increases in the TRPC3 and TRPC6 expression further amplify the CaN-NFAT pathway and contribute to cardiac hypertrophy [51].

We hypothesized that a similar Ca^2+^-dependent CaN-NFAT mechanism underlies the transcriptionally upregulated *Trpc3* gene induced by AβOs in neurons since calcineurin is found to be chronically activated in human AD and in amyloid-based FAD mouse models [52,53]. Soluble AβOs are well known to induce excitotoxic mechanisms via Ca^2+^ overload, which triggers a series of downstream cytotoxic events, including mitochondrial dysfunction, reactive oxygen species (ROS) generation, and a necrosis/apoptosis cascade activation, which ultimately leads to neuronal death [21,22]. For over three decades, excitotoxic Ca^2+^ overload has been largely attributed to the activation of NMDARs as well as many non-glutamatergic Ca^2+^-permeable channels (e.g., voltage-gated calcium channels/VGCCs) [54]. More recently, a specific TRP family member, such as the TRPM2, has been identified to partner with NMDARs for mediating the excitotoxic Ca^2+^ overload in neurons [55]. We therefore speculate that TRPC3 may be another non-glutamatergic Ca^2+^-permeable channel contributing significantly to the Ca^2+^ overload induced by AβOs.

**Figure 4:**
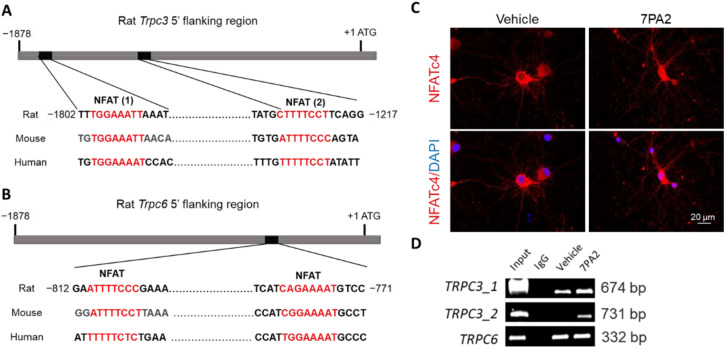
7PA2 treatment induces NFAT4c binding to *Trpc3* gene promoter. (**A**,**B**) Schematic representation of 5′ upstream region of rat *Trpc3* and *Trpc6* genes. Sequences of rat *Trpc3* gene (between –1802 and –1217 bp) and rat *Trip6* gene (between –812 and –771 bp) are aligned with corresponding sequences of mouse and human genes. NFAT sites are shown in red. We identified two NFAT binding sites between positions –1802 and –1217 within *Trpc3* promoter based on motif ([A/T]GGAAA[A/N][A/T/C]N) [57], as well as two NFAT binding sites from promoter Trpc6 [58]. These sites are relatively conserved among humans and rodents. Please note two differently spatially located sites within these two promoters. (**C**) 7PA2 treatment induces NFATc4 nuclear translocation as shown by immunocytochemistry. DAPI (4′,6-diamidino2-phenylindole, blue) staining is used to show colocalization of NFAT immunosignals at 2 h after 7PA2 treatment. (**D**) Representative (bottom) gel images of ChIP-PCR using NFATc4 antibody at 2h after 7PA2 treatment.

To test these hypotheses, we first investigated if the AβO-induced TRPC3 upregulation is mediated as a downstream result of the Ca^2+^ flux. Indeed, the co-treatment of 7PA2 together with a Ca^2+^ chelator EGTA and several NMDAR antagonists completely prevented the TRPC3 upregulation in neurons (Table S5), based on an immunocytochemical analysis with an anti-TRPC3 antibody. Most of these inhibitor compounds are reportedly neuroprotective against excitotoxic conditions in vitro and/or in vivo [56]. We then conducted additional verification experiments to confirm the role of the calcium and calmodulin-dependent serine/threonine protein phosphatase named calcineurin (CaN) in mediating the 7PA2-induced TRPC3 upregulation. Indeed, we found that CaN was overactivated rapidly after exposing neurons to the 7PA2 treatment, as evidenced by a 2-fold increase in the enzymatic activity determined 1 h after the treatment (Figure 3A). Moreover, cyclosporine A (CsA) and tacrolimus (FK506), the two classical calcineurin inhibitors, also completely prevented the AβO-induced TRPC3 upregulation when used in the co-treatment with 7PA2 (Figure 3B,C), with a notable effect detected at concentrations as low as 12.5 nM of FK506 and 1 μM of CsA. We then investigated the kinetics of the nuclear translocation of NFAT3/c4, a predominant isoform in neurons, in response to the 7PA2 treatment (Figure S4). By the chromatin immunoprecipitation ChIP assay, the direct binding of the NFAT was detected on one of the major binding sites in the *Trpc3* promoter region which was induced by the 7PA2 treatment (Figure 4A–C), while no induced binding was detected at the corresponding sites located in the *Trpc6* promoter despite the similarity that two NFAT binding motifs were predicted but at a much closer position (Figure 4D). This finding is consistent with the selectively upregulated *Trpc3* but not the *Trpc6* gene upon the 7PA2 treatment.

We then investigated if the TRPC6 downregulation is at the protein level via the degradation by 7PA2-mediated mechanisms (e.g., proteostatic stress) by testing two widely used inhibitors, Brefeldin A (BFA) and MG132. BFA specifically inhibits vacuolar H^+^-ATPase, a proton pump crucial for the acidification of organelles like lysosomes and endosomes, thus disrupting their functions involving protein degradative machinery like autophagosomes through autophagy. MG132 inhibits the proteasome, a cellular complex responsible for degrading proteins tagged with ubiquitin. By inhibiting the proteasome, MG132 prevents the degradation of ubiquitin-conjugated proteins. Indeed, 7PA2 induced significantly reduced steady-state levels of the TRPC6 proteins, and the co-treatment of 7PA2 with BFA but not MG132 prevented TRPC6 degradation (Figure S5)

These results suggest that the lysosomal, but not the proteasomal, system mediates the AβO-induced protein degradation of TRPC6. Since most of these inhibitor compounds tested could prevent both TRPC3 upregulation and TRPC6 downregulation in 7PA2-treated neurons (Table S5), we reasoned that TRPC6 protein degradation is also the result of an increased cytosolic Ca^2+^ flux. Supporting this theory, we found that the co-treatment with calpeptin, a potent and cell-permeable inhibitor of the Ca^2+^-regulated cysteine protease calpain, also completely prevented TRPC6 degradation (Figure S5C). Therefore, we conclude that TRPC6 is primarily downregulated by AβOs via the facilitated protein degradation involving calpain and the endosomal and lysosomal compartments. Notably, calpain is known to play an important role in the endosomal and lysosomal compartments, particularly in lysosomal permeabilization and cell death [59].

### 3.4. AβOs Upregulate TRPC3 Exclusively in Excitatory Neurons

**Figure 5:**
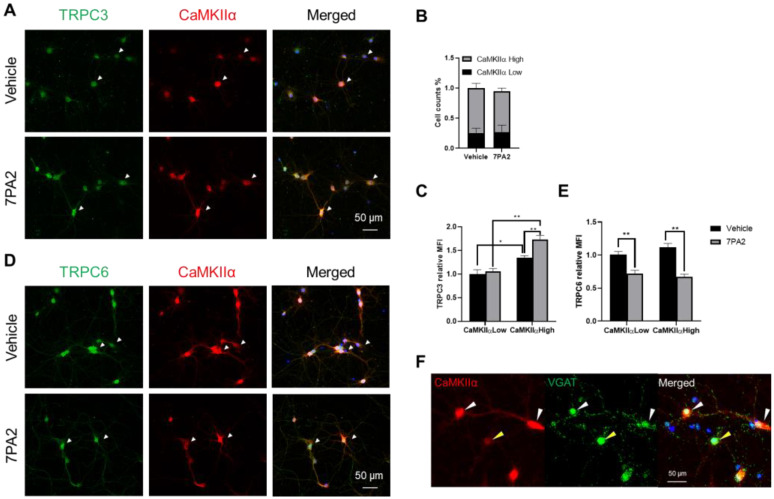
TRPC3 expression is upregulated in mature excitatory neurons upon 7PA2 treatment (4 h). (**A**,**D**) Representative immunocytochemical images of TRPC3 (A, green) and TRPC6 (D, green) co-stained with CaMKIIα (red) and DAPI (blue), taken at 4 h after 7PA2 or vehicle treatment. White arrowheads indicate excitatory neurons identified by high immunosignals of CaMKIIα, which are exclusively colocalized with strong TRPC3 immunosignals. (**B**) Quantification graphs based on number counts of cells expressing high and low levels of CaMKIIα co-localized with 7PA2- upregulated TRPC3. (**C**,**E**) These panels quantify cells of 7PA2-inuduced TRPC3 expression and 7PA2-reduced TRPC6 expression, respectively, in both high and low CaMKIIα populations (gray bars), compared to vehicle group (black bars). * *p* < 0.05; ** *p* < 0.01. (**F**) Representative fluorescent microscopic images of CaMKIIα and VGAT staining, revealing reciprocal staining patterns. White arrowheads indicate two neurons showing strong CaMKIIα immunosignals but weak VGAT, and yellow arrowheads indicate cells with opposite pattern.

TRPC family members (TRPC1-7) are reportedly widely expressed in the CNS and in various cell types, including neurons, glial cells, and endothelial cells [27,29]. Here, we used the primary hippocampal cell culture models to determine the specific cell types in which TRPC3 is upregulated by the 7PA2 treatment, based on the immunocytochemistry of co-staining cells with antibodies against both TRPC3 and the individual marker proteins for each cell type. Our hippocampal neuronal culture typically resulted in over 90 percent neuronal cells and roughly 10 percent glial cells (microglia and astrocytes), as we reported (34). We found that the significantly 7PA2-upregulated TRPC3 was exclusively detected in the excitatory neurons (on both the soma and dendrites) that display high immunosignals of CaMKIIα but not in smaller neurons that show weak CaMKIIα signals (Figure 5A–C). On the contrary, TRPC6-positive immunosignals appear to be equally present in the majority of neurons, including various neuronal subtypes expressing different levels of CaMKIIα (Figure 5D,E).

VGAT (vesicular GABA transporter) is a widely used and reliable marker for identifying GABAergic neurons and the site of GABA release in the presynaptic terminals of these neurons. We also performed double immunocytochemistry using antibodies against CaMKIIα and VGAT, attempting to distinguish between excitatory and inhibitory neurons (Figure 5F). Like CaMKIIα, despite it being the most acclaimed marker for excitatory neurons, we found that both antibodies labeled the majorities of the neuronal cells in our culture model at 14 DIV. However, they do display reciprocal labeling characteristics: the high CaMKIIα-expressing pyramidal neurons often show weak VGAT immunosignals which most likely represent the typical excitatory neurons in the majority (>70–80%, white arrowheads); whist a small percentage of neurons display high VGAT labeling but are co-stained with weak CaMKIIα signals (20–25%). Given the defined neuronal subtypes in the hippocampus, which consists of ~90% excitatory neurons and ~10% GABAergic inhibitory neurons [60], we argued that not all the highly VGAT-positive neurons are inhibitory neurons in our cultures. Nevertheless, the data are clear, concluding that TRPC3 is only upregulated by AβOs in the majority subtype of the excitatory neurons expressing high levels of CaMKIIα.

In primary glial cultures, we also detected increased TRPC3 immunosignals in mature microglia and astrocytes, most notably in the soma (Figure S6A,B). Further support for this conclusion came from the single-nucleus snRNAseq data analysis based on a familial FAD model of APP NL-F-knock-in mice [38], revealing an upregulated Trpc3 gene expression in excitatory neurons but not in inhibitory neurons at a young age (Figure S6C). No significant changes were detected in other cell types (Figure S6D).

### 3.5. Hippocampal CA1-Overexpressed TRPC3 Induces Neuronal Hyperexcitability

**Figure 6:**
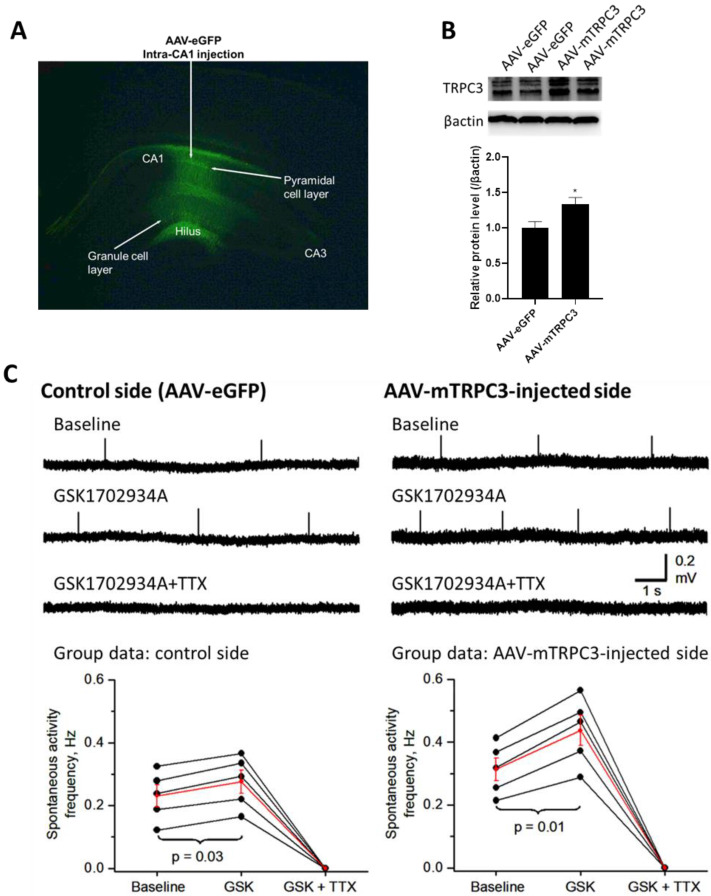
Overactivated TRPC3 channel induces neuronal hyperexcitation in hippocampal CA1 region. (**A**) Representative microscopic image showing green fluorescent protein expression from the intra-CA1-injected AAV-eGFP viruses. (**B**) Western blot shows increased expression of TRPC3 from hippocampi-injected AAV-mTRPC3 and AAV-eGFP. Quantification was based on 3 independent experiments. * *p* < 0.05. (**C**) Increased TRPC3 expression and activation increase neuronal activities in CA1 region in brain slices (N = 5 mice). Left panels display example extracellular recordings of spontaneous neuronal activities in control side CA1 during baseline, during application of 5 μM GSK1702934A (GSK), and upon adding 1 μM tetrodotoxin (TTX). Data are quantified and summarized in bottom graph: each black dot is data point from one mouse, and red dots are the mean of black data points (5 mice, control side CA1). Right panels display same parameter measurements as left panel from AAV-mTRPC3-injected CA1 region. Scale bars apply to both panels. Before and after GSK frequency values in each sample for both groups were compared by paired *t*-test, whereas frequency values during GSK application in two groups were compared by unpaired *t*-test.

To examine the functional aspects of TRPC3, we performed an extracellular recording of the neuronal activity in the CA1 area in hippocampal brain slices from mice that received unilateral intra-CA1 injections of AAV2/9-CAG-mTRP3 (AAV-mTRPC3) to induce TRPC3 overexpression. Intra-CA1-injected AAV-eGFP was used as a control (Figure 6A). The Western blot analysis indicated an increased TRPC3 protein expression in the isolated hippocampal tissue 3 months after the viral injection (Figure 6B), similarly to the AβO-upregulated TRPC3 in cultured neurons. The glass recording electrode tip was inserted just beneath the surface of the CA1 pyramidal neuron layer. With 4 mM KCl in the bathing aCSF, we recorded a baseline spontaneous neuronal activity of 0.231 ± 0.035 Hz in the control side CA1 area; upon the bath application of the TRPC3 agonist GSK1702934A (GSK, 5 μM), this spontaneous neuronal activity was increased to 0.276 ± 0.037 Hz, a modest but significant 20% increase (*p* = 0.03, paired *t*-test) (Figure 6C, left panels). These spontaneous neuronal events had a duration of ~2.5 ms and were completely blocked by 1 μM TTX, indicating that these events were probably conventional fast sodium-dependent neuronal action potentials; action potential-dependent synaptic potentials recorded extracellularly should be much longer, with a duration of at least 10 ms. Under the identical recording conditions, in the AAV-mTrpc3-injected CA1 pyramidal neuron layer, the baseline spontaneous neuronal activity was 0.314 ± 0.036 Hz; this spontaneous activity was increased substantially to 0.437 ± 0.048 Hz (*p* = 0.01, paired *t*-test) by the bath application of 5 μM GSK and was also completely blocked by 1 μΜ TTX (Figure 6C). The basal spontaneous activity frequency showed a clear trend for being higher (36% higher) in the AAV-mTRPC3-injected CA1 (Figure 6C, right panels) than in the control CA1 region (left panels). Although this difference in the basal activity did not reach statistical significance (*p* = 0.1, unpaired *t*-test), the data still indicate a modest basal TRPC3 channel-induced neuronal excitation. Of particular interest, the GSK-induced increase in the spontaneous activity frequency was higher in the AAV-mTRPC3-injected CA1 than in the control CA1 region (mean increase: 39% vs. 20%, *p* = 0.01, unpaired *t*-test). These results indicate that upon activation by a selective exogenous agonist, TRPC3 channels induced statistically stronger CA1 neuronal excitation in the CA1 region overexpressing TRPC3 than in the control CA1 region.

### 3.6. Selective TRPC3 Inhibition Renders Synaptic Protection Against AβOs

Excitotoxicity, the neuronal death caused by overexcitation, and synaptotoxicity, the damage to synapses, are closely linked, with excitotoxicity often leading to synaptotoxicity through mechanisms involving excessive glutamate and calcium overload, ultimately disrupting the synaptic function and structure. Given the overly protective data from the PLC inhibitor compound (Table S4), we speculate that the upregulated TRPC3 plays a pathological role during AβO-mediated excitotoxicity and synaptotoxicity. To test this hypothesis, we utilized both genetic and pharmacological means and focused on testing the effects of TRPC3 suppression on the AβO-induced Ca^2+^ overload and synaptic spine loss.

**Figure 7:**
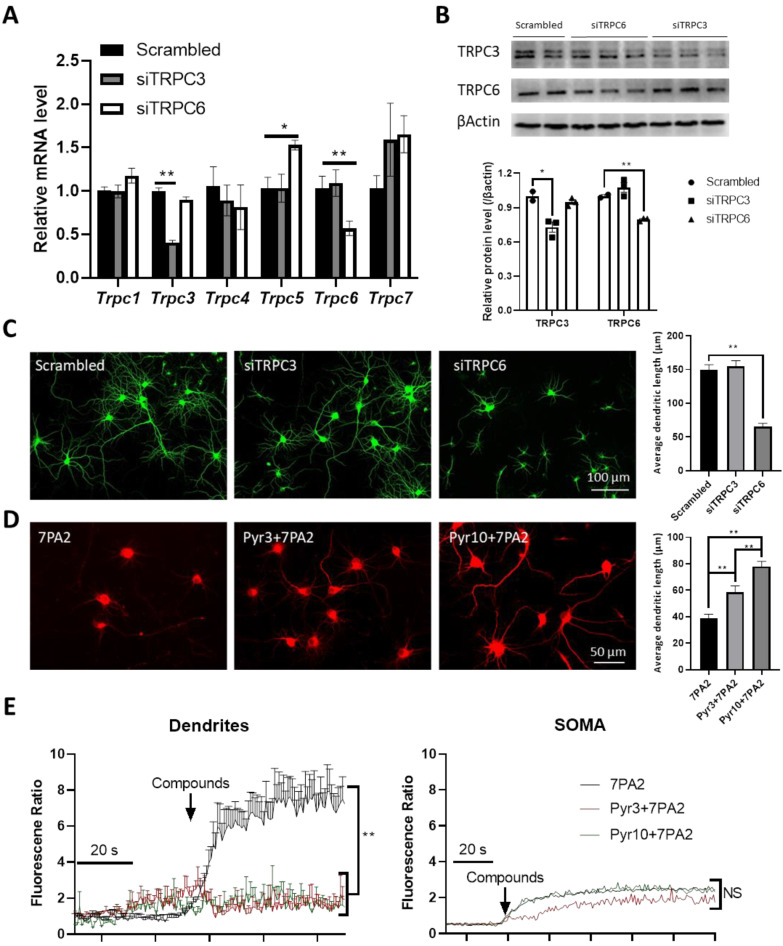
Selective blocking of TRPC3 alleviates 7PA2-induced synaptotoxicity. (**A**,**B**) Specificity of chosen siRNAs. (**A**) Quantified qRT-PCR results from neurons transfected with selected siRNAs against *Trpc3* and *Trpc6* genes. Neurons (5 DIV) were transfected with scrambled or specific siRNAs of Trpc3 and Trpc6 genes (sITRPC3 and siTRPC6). * *p* < 0.05 and ** *p* < 0.01. RT-qPCRs were performed in triplication using specific primers for detecting TRPC family genes. (B) Representative Western blot displays selectively downregulated expression of TRPC3 and TRPC6 proteins in transfected neurons (14 DIV). (* *p* < 0.05 and ** *p* < 0.01). (**C**) Representative MAP2 staining of neurons after being transfected with scrambled or specific siRNAs of Trpc3 and Trpc6 genes, without and with 7PA2 treatment. Quantification was based on 3 independent experiments. ** *p* < 0.01. (**D**) Co-treatment of neurons of 7PA2 with two TRPC3 selective antagonists (Pyr 3 and 10) significantly prevents dendritic loss. Quantification was based on 3 independent experiments. ** *p* < 0.01. (**E**) Co-treatment of neurons of 7PA2 with Pyr3 and 10 completely prevents synaptic Ca^2+^ flux induced by 7PA2. Quantification was based on over 20 neurons in each condition. Arrows indicate when compounds were perfused with and without 7PA2. “NS.” indicates non-significance and ** *p* < 0.01.

We sought to test if the selective downregulation of the *Trpc3* gene expression during the 7PA2 treatment by specific siRNA could protect neurons against AβO-induced synaptic damage. Although the tested siRNAs were selective to downregulate *Trpc3* and *Trpc6* genes and proteins in the mixed neuronal culture without affecting the expression of the remaining TRPC family members (Figure 7A,B), the transfection of the *Trpc3*-selective siRNA did not significantly protect neurons against 7PA2-induced synaptotoxicity, as determined by measuring the dendritic length after the overnight treatment. On the contrary, the *Trpc6*-selective siRNA transfection alone resulted in a severe impairment of the dendritic length, comparable to that from the 7PA2 treatment (Figure 7C). Perhaps the marginal effect of the *Trpc3* gene downregulation (i.e., ~20%) by siRNA at the current transfection efficiency is insufficient to block the 7PA2-induced synaptic toxicity. We then undertook an alternative approach of using pharmacological means. Surprisingly, the Trpc6 expression by specific siRNA at a similar degree of gene downregulation resulted in significant detrimental effects on the MAP2-stained synaptic morphology, indicating a crucial role of TRPC6 in maintaining synaptic functions [61,62,63,64,65].

Lastly, we tested the two pyrazole-based inhibitory compounds of TRPC3, Pyr3, and Pyr10 against AβO synaptic toxicity. As shown in Figure 7D, both compounds render significant protection against the 7PA2-induced synaptotoxicity, with Pyr10 displaying a superior activity in protecting synaptic dendrites. Interestingly, both compounds significantly prevented the Ca^2+^ flux into the dendrites without a significant effect in reducing the Ca^2+^ flux in the neuronal soma (Figure 7E). This finding indicates a mechanistic link between the Ca^2+^ flux in the dendritic compartment to the synaptotoxicity in neurons. Notably, even though both the compounds are selective TRPC3 blockers (compared to other TRPCs such as TRPC6), they differ in their abilities to distinguish between the receptor-operated TRPC3 and the native stromal interaction molecule 1 (STIM1)/Orai1 channels: Pyr3 does not discriminate between Orai and TRPC3 channels, while Pyr10 is a selective inhibitor of the TRPC3 channel [66]. Nevertheless, a critical role of the TRPC3 channel in mediating AβO-induced synaptotoxicity is suggested, which may involve both the TRPC3-mediated receptor- and store-operated calcium entry (ROCE/SOCE) mechanisms.

To confirm if synthetic AβOs can induce TRPC3 upregulation and Ca^2+^ overload in primary hippocampal neurons, we also treated neurons with synthetic Aβ_25–35_ species and found that oligomers displayed the most toxicity over highly aggregated fibrils while monomers had no effect as we reported [34], correlating with their abilities to upregulate TRPC3 expression and impair MAP2-stained dendrites by immunocytochemistry (Figure S7A–D). Consistently, the potencies of these three Aβ states were also demonstrated by their activities in invoking Ca^2+^ flux (Figure S7E). The Aβ-induced synaptotoxicity appeared to be dependent on the degrees of the evoked Ca^2+^ flux response, and the severity of these two events is tightly correlated, suggesting their mechanistic link.

## 4. Discussion

We report here for the first time that the TRPC3 and TRPC6 expression are differentially regulated by soluble oligomeric AβOs in excitatory neurons. More interestingly, they are regulated primarily at the levels of the *Trpc3* gene transcription and TRPC6 protein degradation, respectively. The upregulated TRPC3 expression has also been supported by two separate cohorts of post-mortem AD brain specimens. Additionally, bulk RNAseq and snRNAseq confirmed the upregulated *Trpc3* gene in a familial AD mouse model (e.g., 5xFAD), as we recently reported [67], and in a second APP-KI model at a young age (Figure S6C) [38]. These findings appear to corroborate our data collected from cultured primary hippocampal neurons (Figure 5) showing selectively upregulated TRPC3 in large pyramidal excitatory neurons. Although the quantification of the upregulated TRPC3 immuno-DAB signals using two different approaches (Figure 1F) reveals a slightly larger increase in TRPC3 in pyramidal neurons than the total cells in the AD versus NC samples, the increase in the TRPC3 expression in the latter case (i.e., mixed brain cells) is equally significant (*p* < 0.01). Notably, this result was based on a limited number of human brain specimens with the majority of AD samples from female patients (Table S4). Given the notions that, compared to male patients, female AD patients often display a more severe pathology, especially in terms of neurofibrillary tangles. Furthermore, female patients also tend to develop more severe clinical symptoms even with the same degree of AD pathology as male patients [68,69]. It thus remains to be determined if TRPC3 is more upregulated in female AD brains than males. The cell-type specificity of the upregulated TPPC3 in AD requires further investigation based on a larger cohort of human specimens with a better sex balance. Below we will focus on comparing distinct dysregulations and functional roles of these two closely related TRPC members (i.e., TRPC3 and 6) in the context of AD.

### 4.1. Differential Regulation of TRPC3 and TRPC6 in AD

We demonstrate here that the *Trpc3* gene is transcriptionally upregulated in AD as determined by RT-qPCRs from both primary cultured mature neurons and in human AD brains. The ChIP result indicates the binding of NFATc4 to the *Trpc3* gene promoter shortly after the 7PA2 treatment, which could be prevented by the two CaN inhibitor co-treatments. Calcineurin/CaN-coupled NF-AT nuclear translocation is a well-established transcriptional pathway downstream of Ca^2+^ signaling, where Ca^2+^ activation leads to CaN activation, the dephosphorylation of NFAT, and, subsequently, the NFAT’s translocation to the nucleus, where it regulates gene expression [70]. Although this pathway is required for the expression of Ca^2+^-dependent genes under both physiological (e.g., neurotransmission and synaptic plasticity upon neuronal activity) [71] and pathological conditions, the latter was more widely reported. Overactivated CaN and NFAT signaling in both neurons and astrocytes has received much attention during the AD pathogenesis and is currently considered as one viable therapeutic strategy [72]. The CaN-NFAT signaling has also long been implicated in regulating the hypertrophic growth of the myocardium and contributing to heart failure [73]. The overactivation of TRPC3 and TRPC6 channels, coupled with the CaN-NFAT signaling pathway, plays a crucial role in the development of myocardial hypertrophy by enhancing the Ca^2+^ influx and promoting pathological cardiac remodeling [74]. Notably, it is the NFAT that mediates the upregulated *Trpc3* and *Trpc6* gene promoters which, in turn, contributes to the activation of the CaN-NFAT signaling pathway. Of particular interest, we only detected increases in the NFAT binding to the *Trpc3*, but not *Trpc6*, gene promoter upon the 7PA2 treatment of the neuronal culture. It remains as a paradox why the nuclear translocated NFAT binding excludes the binding sites in the Trpc6 gene promoter in neurons. We speculate that neurons display a different promoter architectural landscape, encompassing the diverse way the *Trpc* 3 and *Trp6* gene promoters are structured from the other cell types (e.g., cardiomyocytes) that determines their gene expression being subjected to differential regulatory modes. The latter can include diverse nucleosome positioning, regulatory element combinations, and the presence of specific motifs, impacting gene regulation and expression variability. On the other hand, distinct coactivators may also play a crucial role in the promoter regulation of the neuronal *Trpc3/6* genes by interacting with different transcription factors and influencing their chromatin structure, ultimately modulating gene expression in a context-specific manner [75].

Although we observed a decline but a non-statistical significance of the *Trpc6* mRNA levels in both human AD and in 7PA2-treated neurons by RT-qPCRs, our preliminary results presented in Figure S5 suggest protein degradation is primarily responsible for the TRPC6 downregulation by AβOs. Indeed, the preventive data from co-treatments with the calpain inhibitor confirmed the Ca^2+^ signaling as the initiating factor. In fact, TRPC6 protein degradation was originally reported in the context of ischemic stroke via calpain-mediated proteolysis involving the calpain cleavage of the N-terminal domain of TRPC6 at Lys16 [76]. On the other hand, we also found that the BFA-sensitive endosome–lysosomal, but not the proteasomal, pathways mediate the TRPC6 protein degradation upon 7PA2 treatments, which is consistent with a prior report [77].

### 4.2. Potential Implications of the Upregulated TRPC3 and Downregulated TRPC6 in AD: Gain-of-Function vs. Loss-of-Function in Ca^2+^ Dysregulation and Hyperexcitability

*Neuroprotective TRPC6 vs. potentially synaptotoxic TRPC3 upon upregulation.* A TRPC6 deficiency or increased activity due to gain-of-function mutations has been associated with a multitude of diseases, such as kidney disease, pulmonary disease, and neurological disease [78]. Among the TRPC family, TRPC6 has been the best-characterized member for its multiple neuro- and synaptoprotective actions. TRPC6 plays a role in synapse development, including nerve growth cone guidance [61], neurite outgrowth [62], the formation of excitatory synapses [63], and mushroom spine morphology changes [64]. Its neuroprotective effect has been widely reported against ischemic stroke [79,80], involving mechanistic actions on multiple cell types across neurovascular units [81], despite a conflicting report [82] with unknown reasons. Notably, TRPC6 is found to be a crucial factor coupling with Orai 2 in forming the stromal interaction molecule 2 (STIM2)-regulated neuronal-store-operated Ca^2+^ influx (nSOC) channel complex in the hippocampal synapse; the resulting Ca^2+^ influx is critical for the long-term maintenance of mushroom spines, the strong and stable synapses for memory storage (64). TRPC6 variants (i.e., loss-of-function mutations) are candidate risk genes for Autism Spectrum Disorder (ASD) [83]; TRPC6-mediate neuronal SOCE has been demonstrated in a human iPS model to be crucial in counteracting neuronal hyperexcitability in ASD [84].

On a separate note, although it is believed that TRPC3 and TRPC6 can form heterotetramers under certain pathological conditions [85], based on the data we collected and the literature, we argue against the TRPC3/6 heterotetramers formed in AD-type neurons. Furthermore, the recent high-resolution proteomics of rodent brains also suggests that TRPC3, C6, and C7 preferentially form homomers, and TRPC1-, C4-, and C5-containing channels are mostly heteromers with defined stoichiometries for each subtype [86]. In fact, despite the fact that both genes are reported to be widely expressed in the CNS, they display differential expressional profiles along the hippocampal subregions and the related areas. For example, TRPC3 is reportedly expressed in the CA1 and CA3 regions [87], which was confirmed by us (Liao, unpublished data), while TRPC6 is localized in the molecular layer and in interneurons of the dentate gyrus/DG in the hippocampal formation [88]. It is worth pointing out that immunocytochemistry on our neuronal culture derived from isolated hippocampal tissue indicates that TRPC3 and TRPC6 are expressed in almost all the neuronal types, and they seem to be co-expressed in the majority of the hippocampal excitatory neurons. Of particular interest, TRPC3 was reportedly to be the only member from the family localized in the inner membrane of the mitochondria in non-neuronal cells, in addition to the plasma membrane [89]. This notion is confirmed in excitatory neurons where TRPC3 and TRPC6 can be spatially separated in different intracellular subcompartments (e.g., endoplasmic reticulum/ER vs. mitochondria) and exert distinct roles, such as in intracellular Ca^2+^ handling. A further investigation to fully understand the potentially distinct mechanisms of TRPC3 versus TRPC6 in mediating neuronal Ca^2+^ signaling will be instrumental for the future therapeutic development of novel agents in combating AD.

*Upregulated TRPC3 and neuronal hyperexcitability*. Hyperexcitability reflected by excessive and uncontrolled neuronal activity is not only the key hallmark of seizures but is also implicated in early stages of AD [90]. Our findings of chronically upregulated TRPC3 in human AD brains (Figure 1) and in 5xFAD mice [63] implicate that an overactivated TRPC3 channel may contribute to hyperexcitability. Indeed, we provided direct evidence supporting this theory in Figure 6. Moreover, we also reported a Pyr3-based compound JW65 in preventing both seizures [91] and AD-related long-term memory deficits [63]. Similarly, overactivated TRPC3 channel activities in Purkinje neurons, as shown by the gain-of-function mutation (T635A) in the *Trpc3* gene, account for the impaired dendritic development and survival underlying cerebellar ataxia [92].

Thus far, we have reviewed the evidence implicating the loss of function of TRPC6 versus the potential “gain of function” of overactivated TRPC3 in several neurodegenerative conditions, including stroke, seizures, and AD [93,94,95], painting a fascinating picture of the distinct pathological roles displayed by these two closely related members in neurodegeneration. Strikingly, despite the well-characterized physiological role of TRPC3 in manifesting BNDF-mediated synaptic transmission and dendritic maturation via its ionic Ca^2+^ current [96,97,98,99], our work reveals a negative role of TRPC3 in the AD pathogenesis, presumably via overactivated channel activities. Future studies are warranted to provide experimental support to distinguish these two facets of TRPC3 in physiology and pathology.

## Figures and Tables

**Table 1:** A summary of the oligonucleotide primers used in the quantitative PCR analysis, ChiP, and siRNA.

	Forward	Reverse
*hTrpc3*	TGCAGAAGGAGAAGGCTTC	ACGTGTTGGCTGATTGAGA
*hTrpc6*	GTGGCCTATGTCAAGTATAA	GGCAGTAGATAAAGAGGAAT
*hβActin*	CCCGCGAGTACAACCTTCT	CGTCATCCATGGCGAACT
*rTrpc1*	CACGATTTCCCTCTCAGC	AGTTCCTGAACACCGTTTGG
*rTrpc3*	CCACATGCAGTGAGACTTTGACTC	AGGCCAACCTTGGGATCATTT
*rTrpc4*	AAGGATTAGCTTCACGGGGTG	CCTCCTCCTGGGCGTGTTTC
*rTrpc5*	TGAGTCGTCAGGCAAACGGTC	AGAAATTTGGAATTTTGGGAAGTC
*rTrpc6*	AGAAATTTGGAATTTTGGGAAGTC	TCCTTATCAATCTGGGCCTGC
*rTrpc7*	TCCCTTTAACCTGGTGCCGAGTC	TTCAGCATGCCCATTTCCAGG
*rβActin*	CCCGCGAGTACAACCTTCT	CGTCATCCATGGCGAACT
*Chip_Trpc3_1*	CGTTGGTTACAGCCAACCTC	GCCCTTACTGGTGGGGTATT
*Chip_Trpc3_2*	GGCTGTCAGGGAACTGTCTC	GAAATCACCCCCTGCTGGAA
*Chip_Trpc6*	TTAGGACAAGCAGAGCCACG	GGGCTAACTGCTCCCAAAGT
siTRPC3	UCAUCUUCCUGGGUCUGCUUGUGUU	AACACAAGCAGACCCAGGAAGAUGA
siTRPC6	GGAGCUCAGAAGAUUUCCAUUUAAA	UUUAAAUGGAAAUCUUCUGAGCUCC

## Data Availability

Raw and processed snRNA-seq data used in this study are accessible [38].

## Supplementary Materials

The following supporting information can be downloaded at https://www.mdpi.com/article/10.3390/cells14110843/s1: Figure S1. 7PA2 CM induces neurotoxicity and synaptoxtoxicity in mature hippocampal neurons. (A) Representative immunoblots (10–20% SDS-Tricine gels) (34) showing lower n-AβOs detected from the 7PA2 CM after TCA precipitation with a final concentration of 10% and washed with ice-cold acetone once (lanes 2 and 3) compared to the flow-through (Lane 1), which were largely undetectable after immunoprecipitation with BAM-10 and 4G8 (1:1000) (lane 4). (B) Quantified cell death based on Annexin V fluorescence determined overnight after 7PA2 treatment (n = 3 experiments). Annexin V is a Ca^2+^-dependent phospholipid-binding protein that binds to phosphatidylserine (PS) at high affinity and thus detects the PS which are only present in the outer leaflet of the cellular membrane during early apoptotic process. It is a commonly used method to detect early apoptotic cell events using fluorescently labeled annexin V protein in staining live cells. (C) Representative microscopic images (20× magnification from KEYENE microscope) of the MAP2-stained neurons treated with 7PA2 for various time points. Please see the Methods for details regarding quantification. (D) Representative MAP2 stained image (960 × 720 image size) illustrating the strategy we used to quantify the average dendritic length: representative dendrites were selected and marked by the white dotted lines for the ImageJ to quantify the mean length (averaged 2–3 dendrites per neurons from 15 to 20 neurons per group). (E) Representative field image of glial cells in our neuronal culture which typically contains <10% mixed GFAP-positive astrocytes (red arrow) and occasionally detected IBA1-positive microglia (green arrow). Figure S2. Aβ-depleted 7PA2 CM diminishes neurotoxicity and synaptoxtoxicity in mature hippocampal neurons, as determined by comparative effects from the 7PA2 CM before and after immunodepletion by anti-Aβ antibodies in (detected by DHE), apoptosis (Annexin V), and dendritic loss (MAP2). 7PA2 CM immunodepletion was performed as described for Figure S1A. Quantification was based on 3 independent experiments. ** *p* < 0.01. Figure S3. Altered expressional profiles of the TRPC family members upon 7PA2 treatment. (A) Representative microscopic images of the immunostained TRPC members taken 16 h after 7PA2 treatment. Total MFI (B) and neuronal MFI (C) quantification based on 4 independent experiments shows significantly increased TRPC3 but reduced TRPC4, 5, and 6 proteins. (D) Quantified expression of the genes encoding for the TRPC family after 7PA2 treatment determined by RT-qPCR, based on 3 independent experiments. * *p* < 0.05 and ** *p* < 0.01, respectively. Figure S4. The time course of the 7PA2-induced NFAT nuclear translocation event in mature hippocampal neurons. Immunocytochemistry was performed using anti-NFATc4 antibody at the indicated time points, counterstained by DAPI. White arrowheads mark the neurons with nucleus-translocated NFAT as indicated by overlapped red and blue fluorescence. Quantification was based on 2 independent experiments of the NFAT immunosignals in the nuclei. ** *p* < 0.01. Figure S5. 7PA2 treatment induces TRPC6 protein degradation via the lysosome-endosome pathway. (A, D) Representative images of TRPC6 immunosignals detected at 16 h after various treatments in primary hippocampal neurons. Neurons were co-treated with BFA (5 µg/mL), MG-132 (5 µM), calpeptin (20 µM), and 7PA2. (B,E) Representative immunoblots of TRPC6 protein with lysates prepared from primary cortical neurons at 16 h after the treatments. (C and F) Quantifications of the WBs from the experiments of panels B and D, based on 3 independent experiments. * *p* < 0.05, and ** *p* < 0.01, respectively. Figure S6. 7PA2 cell treatment increases TRPC3 expression in mature microglia (A) and astrocytes (B): white arrowheads indicate cells with increased TRPC3 immunosignals (Inset in A), and the cell body of astrocytes (right panel B). (C) Selectively upregulated *Trpc3* gene detected in excitatory neurons by single-nucleus RNAseq analysis. Dot plots showed that the average expression of the Trpc3 gene increased in the excitatory neurons, but not in the inhibitory neurons, of App^NL-G-F/wt^ mice compared to control mice. (D) The same snRNAseq data on various cell types. Dot plots show that the average expression of *Trpc3* in oligodendrocyte precursor cells/OPCs, oligodendrocytes, microglia, and astrocytes was not significantly altered in the App^NL-G-F/wt^ mice compared to control mice (NS: non-significant). Endothelial cells were not identified in our experimental samples [38]. Figure S7. Aβ_25-35_ oligomers are synaptotoxic to neurons. (A) Representative immunostained TRPC3 images of the hippocampal neurons treated with Aβ_25-35_ monomers, soluble oligomers and fibrils, all at 10 µM (4 h) based on the monomeric starting peptide concentrations. (C) Representative immunostained MAP2 images of the treated hippocampal neurons with Aβ_25-35_ species at 10 µM for 16 h. (B, D) Quantification of results from experiments in panels A and B, respectively, based on 4 experiments. * *p* < 0.05; ** *p* < 0.01. (E) Calcium imaging of the neurons with 7PA2 CM or Aβ_25-35_ species (10 μM). Arrow indicates when the Aβ was perfused. Black arrow indicates when the perfusion stated with 7PA2 or synthetic Aβ_25-35_ species (10 μM). Tables S1–S4: lists of the details of the human specimens used. Table S5. Effects of Ca^2+^ chelator and inhibitor compounds in preventing TRPC3 upregulation and TRPC6 downregulation induced by 7PA2 treatment, as determined by immunocytochemistry based on at least four replicated experiments. “+” indicates positive effect in preventing 7PA2-induced altered expressions of TPPC3 and/or TRPC6; while “-” indicates undetectable effect.
