# Supplementary material for: Soluble β-Amyloid Oligomers Selectively Upregulate TRPC3 in Excitatory Neurons via Calcineurin-Coupled NFAT
Source: Cells. 2025 Jun 4;14(11):843. doi: 10.3390/cells14110843 (PMC12155350; doi:10.3390/cells14110843)
Supplement: Supplementary file 1 [file cells-14-00843-s001.zip › cells-3597093-supplementary.pdf]

Table S1. Human brain cortical tissue samples used in RT-qPCR analysis.

	ID	Age	Gender	Clinical Diagnoses
NC	3216	79	M	Congestive Heart Failure, Hypertension
NC	3221	90	M	COPD, dizziness, etiology unknown, Migraine
NC	3236	67	M	CA, Esophagus
NC	3276	54	M	Coronary heart disease, Peripheral vascular disease, End stage renal disease
NC	3371	52	M	CA, Lung
NC	3406	72	F	Congestive Heart Failure, CA, Breast. cancer, Tuberous sclerosis
AD	4007	81	M	AD, Parkinson's disease, Dementia
AD	4012	86	F	AD, Parkinson's disease, Dementia, Hypertension, Pneumonia
AD	4023	72	M	AD, Dementia, Congestive heart failure, Atrial fibrillation, Hypertension, Alcohol abuse, Deep Venous thrombosis,
AD	4043	80	F	AD, CA, colon, Hypothyroidism, Depression
AD	4091	88	F	AD, Dementia, Stroke, Migraine, Crohn's disease, Atrial fibrillation, Pulmonary emphysema, Hypertension, Hypothyroidism
AD	4098	68	F	AD, Dementia
AD	4125	61	F	AD, Atrophy, Posterior Cortical, Depression, Acoustic neuroma

COPD: Chronic Obstructive Pulmonary Disease

CA: cancer/carcinoma

Table S2. Human brain hippocampal tissue samples used in Western blot analyses.

	ID	Age	Gender	Clinical Diagnoses
NC	3298	79	M	CA, Prostate, Renal Failure, Acute, Diabetes Type I
NC	3406	72	F	Congestive Heart Failure, CA, Breast, Tuberous sclerosis
NC	3401	82	M	Congestive Heart Failure
NC	3465	93	F	Von Willebrand's disease
NC	567	79	M	Depression, Major, Suicide, overdose, Alcohol Abuse
NC	600	37	M	Depression, Major, Alcohol Abuse, Hypertension
NC	620	39	M	Depression, Major, Suicide, gunshot, head
AD	4038	88	F	AD, Dementia, Congestive heart failure, Hypertension, Depression, Hypotension, Chronic urinary tract infection
AD	4083	76	M	AD, Dementia, Hypertension, Coronary heart disease, Hypotension, Aggressive behavior
AD	4091	88	F	AD, Dementia, Stroke, Migraine, Crohn's disease, Atrial fibrillation, Pulmonary emphysema, Hypertension, Hypothyroidism
AD	4098	68	F	AD, Dementia
AD	4125	61	F	AD, Atrophy, Posterior Cortical, Depression, Acoustic neuroma

AD	4023	72	M	AD, Dementia, Congestive heart failure, Atrial fibrillation, Hypertension, Alcohol abuse, Deep Venous thrombosis
AD	4012	86	F	AD, Parkinson's disease, Dementia, Hypertension, Pneumonia
AD	4004	94	F	AD, Paranoia, Macular degeneration, Depression, Osteoporosis
AD	4007	81	M	AD, Parkinson's disease, Dementia

NC: non-AD controls

AD; by neurological diagnosis

Table S3. Human brain cortical samples provided in formalin (in year 2020) used in immunohistochemistry.

	Sample ID	Age	Race	Gender	Braak	B-amyloid levels	PMI (hr)	Diagnosis
NC	5293	88	white	M	1	Low	2.67	VaD+ARTAG
NC	5077	80	white	F	3	Low	5	VaD+ARTAG
NC	5062	92	white	M	2	Low	2	Normal+multiple infarcts
NC	1304	81	white	M	0	Low	4.83	Normal+ARTAG
AD	5272	78	white	F	6	High	5.5	ADNC+CVD
AD	1068	92	white	F	6	High	2	ADNC+DLB+CVD
AD	5079	85	white	F	6	High	6	ADNC +CVD
AD	5371	93	white	F	5	High	4.8	ADNC +CVD
AD	5353	98	white	F	2	High	1.75	ADNC +CVD

NC: non-AD cases

VaD: Vascular Dementia

ARTAG: Arteriosclerosis-Atherosclerosis

CVD: Cardiovascular Disease

DLB: Dementia with Lewy Bodies

ADNC: Alzheimer's Disease with confirmed Neuropathologic Changes

Table S4. Summary of the genders of the human specimens used in studies presented in Figure 1.

	Non-AD controls (Male / Female)	AD (Male / Female)
Western blot	5 / 2	3 / 6
RT-qPCR	5 / 1	2 / 5
Immunohistochemistry	3 / 1	0 / 5
Total	13 / 4	5 / 16

Table S5. Effects of Ca^{2+} chelator and Ca^{2+}-dependent pathway inhibitor compounds in 7PA2-induced TRPC3/6 changes. Effects on TRPC3 upregulation and TRPC6 downregulation induced by 7PA2 treatment was determined by immunocytochemistry based on at least four replicated experiments. "+" indicates positive effect in preventing 7PA2-induced altered expressions of TPPC3 and/or TRPC6; while "-" indicates undetectable effect.

		Inhibitors	Concentration	TRPC3	TRPC6
				4 hr	4 hr
Calcium chelator		EGTA	1 mM	+	+
NMDARs	Competitive	AP V	50 μM	+	-

	Non-competitive	MK801	10 μM	+	+/-
		Memantine	1 μM	+	+
	NR2B	Ifenprodil	10 μM	+	+/-
AMPAR		CNQX	10 μM	+/-	+
VGCCs	L-type	Nifedipine	5 μM	+	+/-
		Verapamil	5 μM	+/-	+/-
	T-type	ML218	5 μM	+	-
Calcineurin		FK506	500 nM	+	+/-
		CsA	10 μM	+	-
		2APB	10 μM	+	-
PLC		U73122	5 μM	+	-
TRPC3/ORIA 1		Pyr3	1 μM	+	-
TRPC3		Pyr10	1 μM	+	-

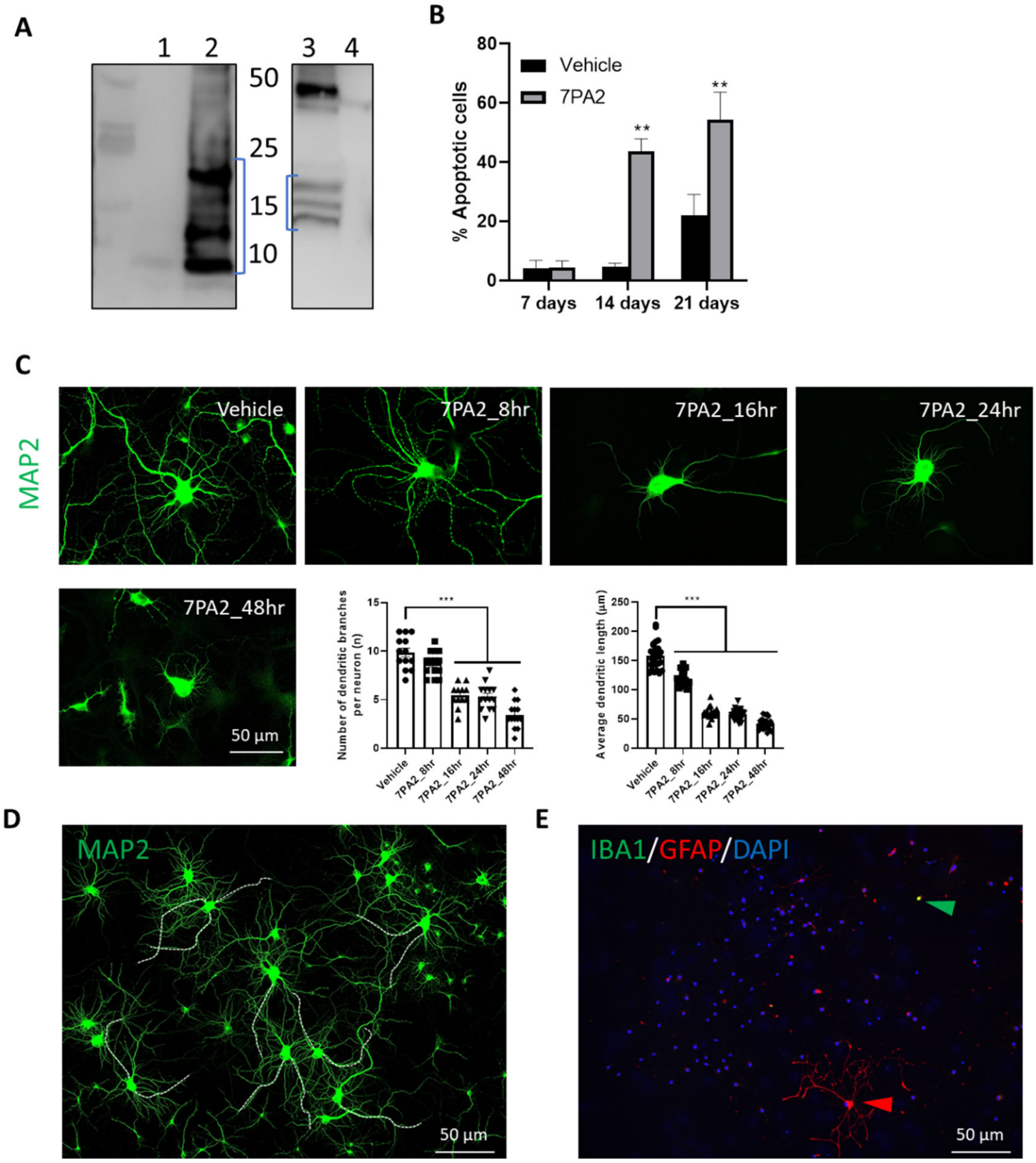

Figure S1. 7PA2 CM induces neurotoxicity and synaptoxtoxicity in mature hippocampal neurons. (A) Representative immunoblots (10-20 % SDS-Tricine gels) (34) showing lower n-AβOs detected from the 7PA2 CM after TCA precipitation with a final concentration of 10% and washed with ice-cold acetone once (lanes 2 and 3) compared to the flow-through (Lane 1), which were largely undetectable after immunoprecipitation with BAM-10 and 4G8 (1:1000) (lane 4). (B) Quantified cell death based on Annexin V fluorescence determined overnight after 7PA2 treatment (n=3 experiments). Annexin V is a Ca^{2+}-dependent phospholipid-binding protein that binds to phosphatidylserine (PS) at high affinity and thus detects the PS which are only present in the outer leaflet of cellular membrane during early apoptotic process. It is a commonly used method to detect early apoptotic cell events using fluorescently labeled annexin V protein in staining live cells. (C) Representative microscopic images (20 x magnification from KEYENE microscope) of the MAP2-stained neurons treated with 7PA2 for various time points. Please see the Methods for details regarding quantification. (D) Representative MAP2 stained image (960x720 image size) illustrating the strategy we used to quantify the average dendritic length: representative dendrites were selected, and marked by the white dotted lines for the ImageJ to quantify the mean length (averaged 2-3 dendrites per neurons from 15-20 neurons per group). (E) Representative field image of glial cells in our neuronal culture which typically contain <10 % mixed GFAP-positive astrocytes (red arrow), and occasionally detected IBA1-positive microglia (green arrow).

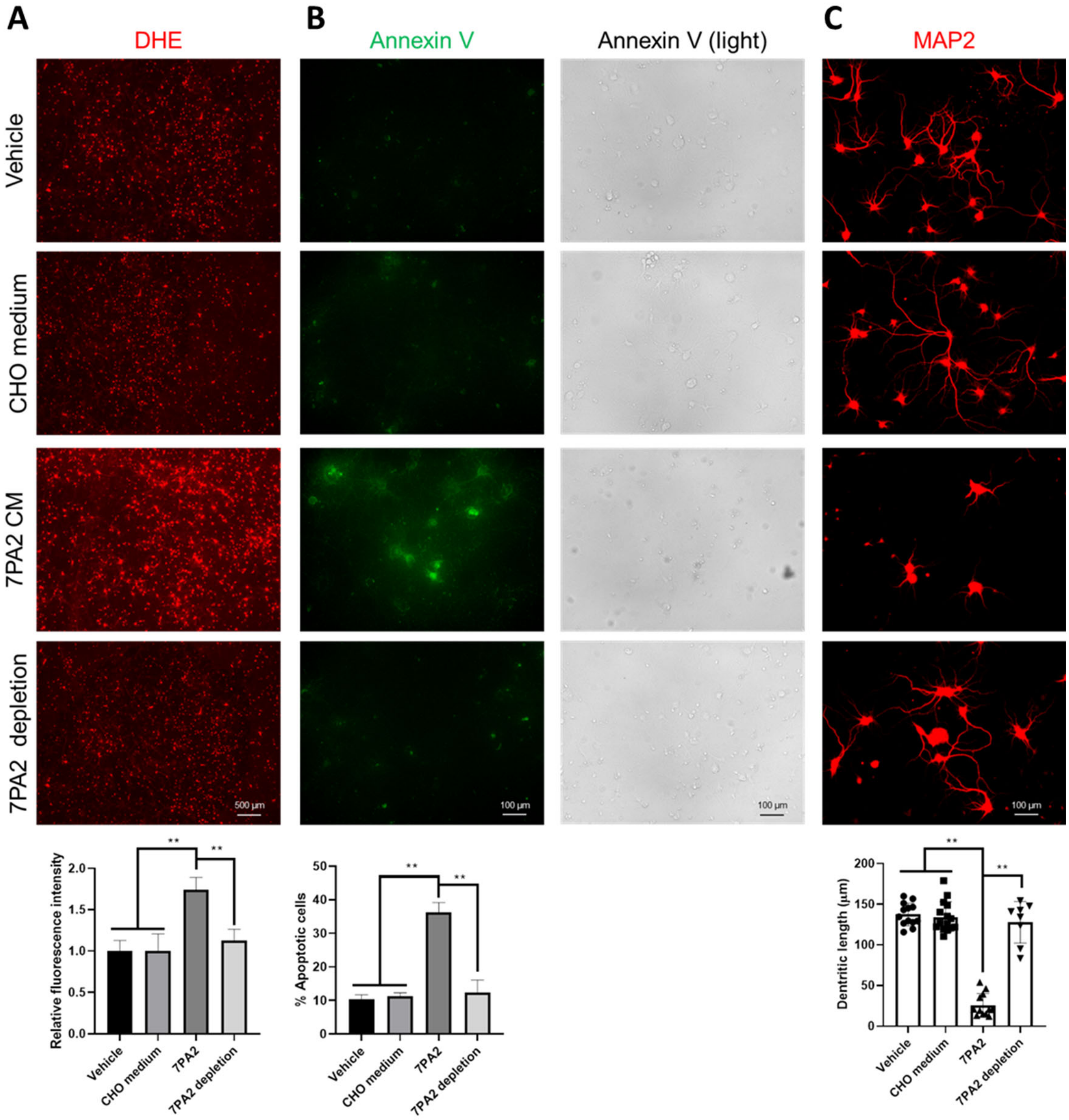

Figure S2. Aβ-depleted 7PA2 CM diminishes neurotoxicity and synaptoxtoxicity in mature hippocampal neurons, as determined by comparative effects from the 7PA2 CM before and after immunodepletion by anti-Aβ antibodies in (detected by DHE), apoptosis (Annexin V) and dendritic loss (MAP2). 7PA2 CM immunodepletion was performed as described for the SFigure 1A. Quantification was based on 3 independent experiments. ***p*<0.01.

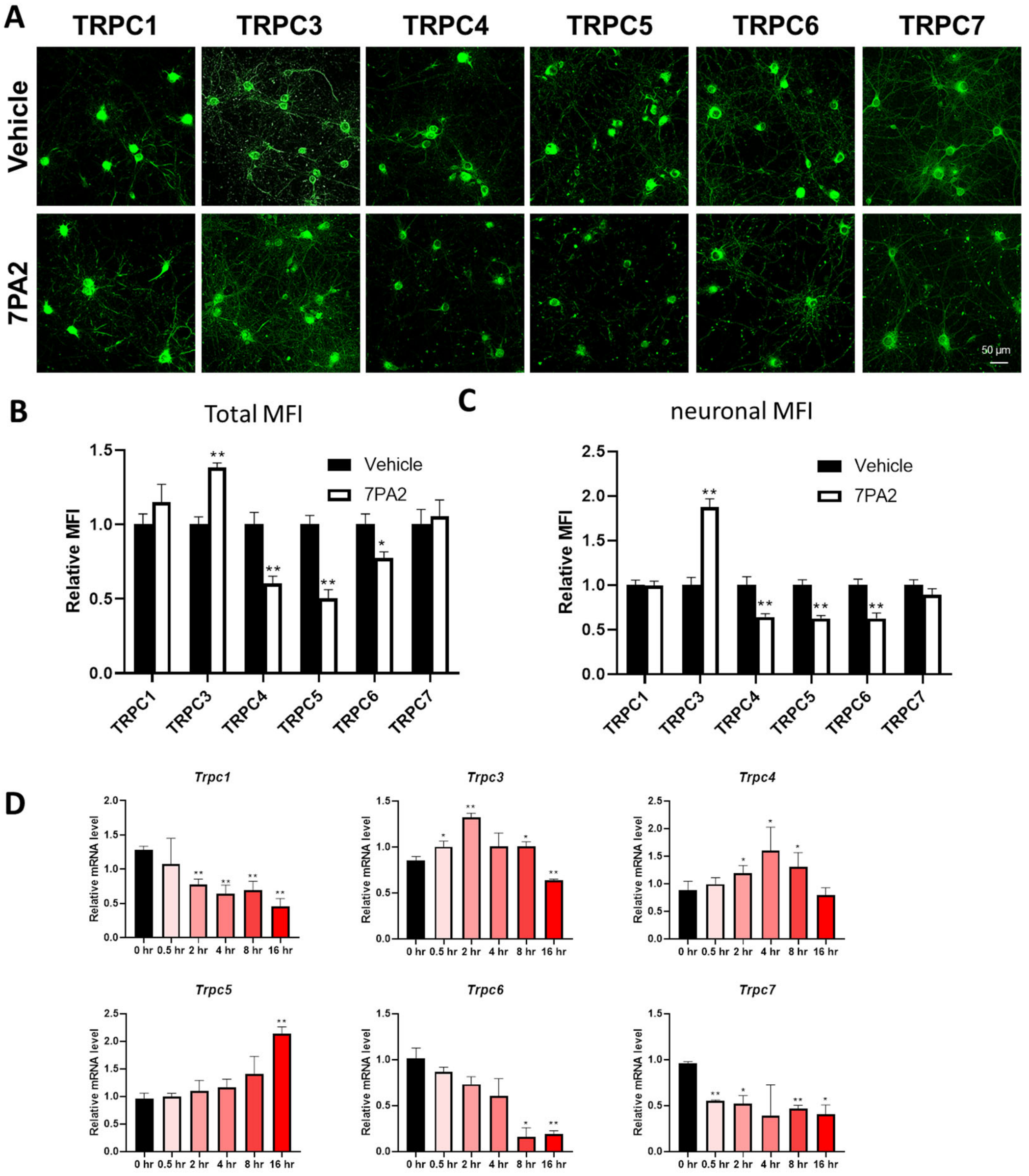

Figure S3. Altered expressional profiles of the TRPC family members upon 7PA2 treatment. (A) Representative microscopic images of the immunostained TRPC members taken 16 hr after 7PA2 treatment. Total MFI (B) and neuronal MFI (C) quantification based on 4 independent experiments shows significantly increased TRPC3 but reduced TRPC4, 5, and 6 proteins. (D) Quantified expression of the genes encoding for the TRPC family after 7PA2 treatment determined by RT-qPCR, based on 3 independent experiments. *p<0.05 and **p<0.01, respectively.

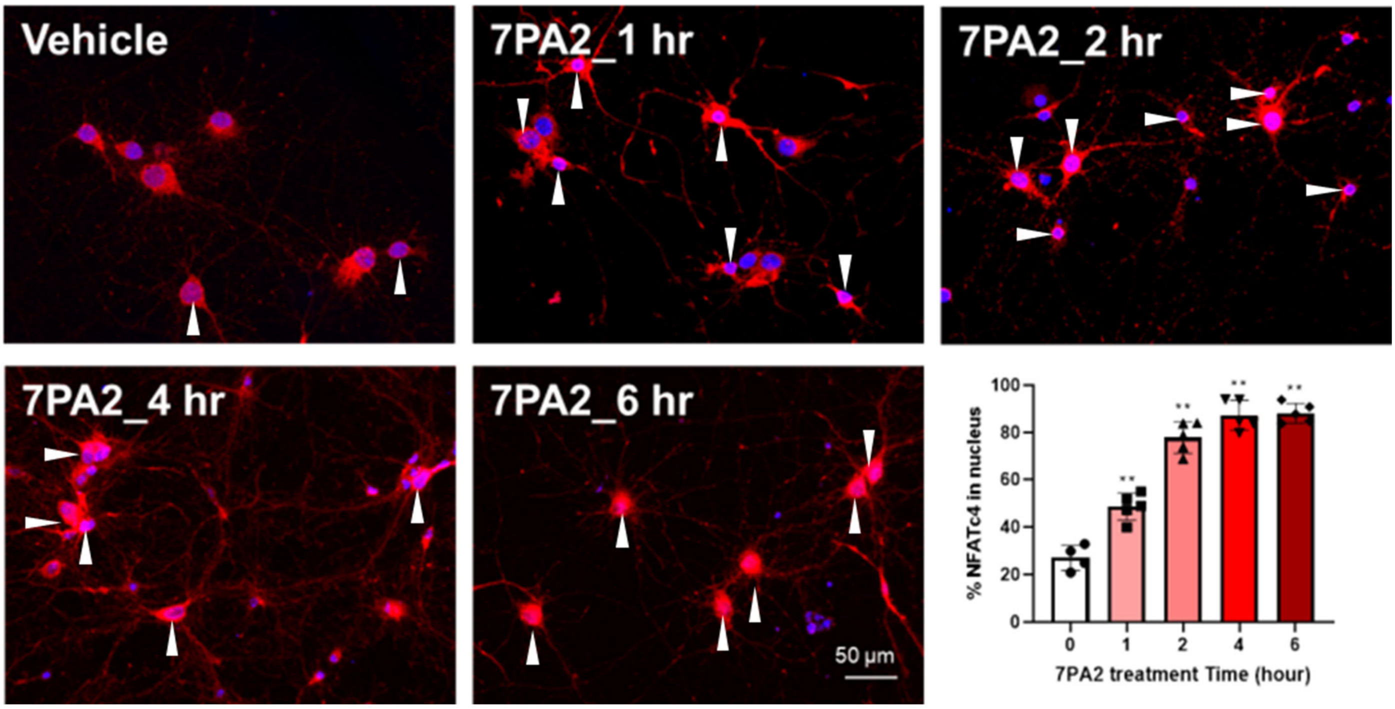

Figure S4. The time course of the 7PA2-induced NFAT nuclear translocation event in mature hippocampal neurons. Immunocytochemistry was performed using anti-NFATc4 antibody at the indicated time points, counterstained by DAPI. White arrowheads mark the neurons with nucleus-translocated NFAT as indicated by overlapped red and blue fluorescence. Quantification was based on 2 independent experiments of the NFAT immunosignals in the nuclei. **p<0.01.

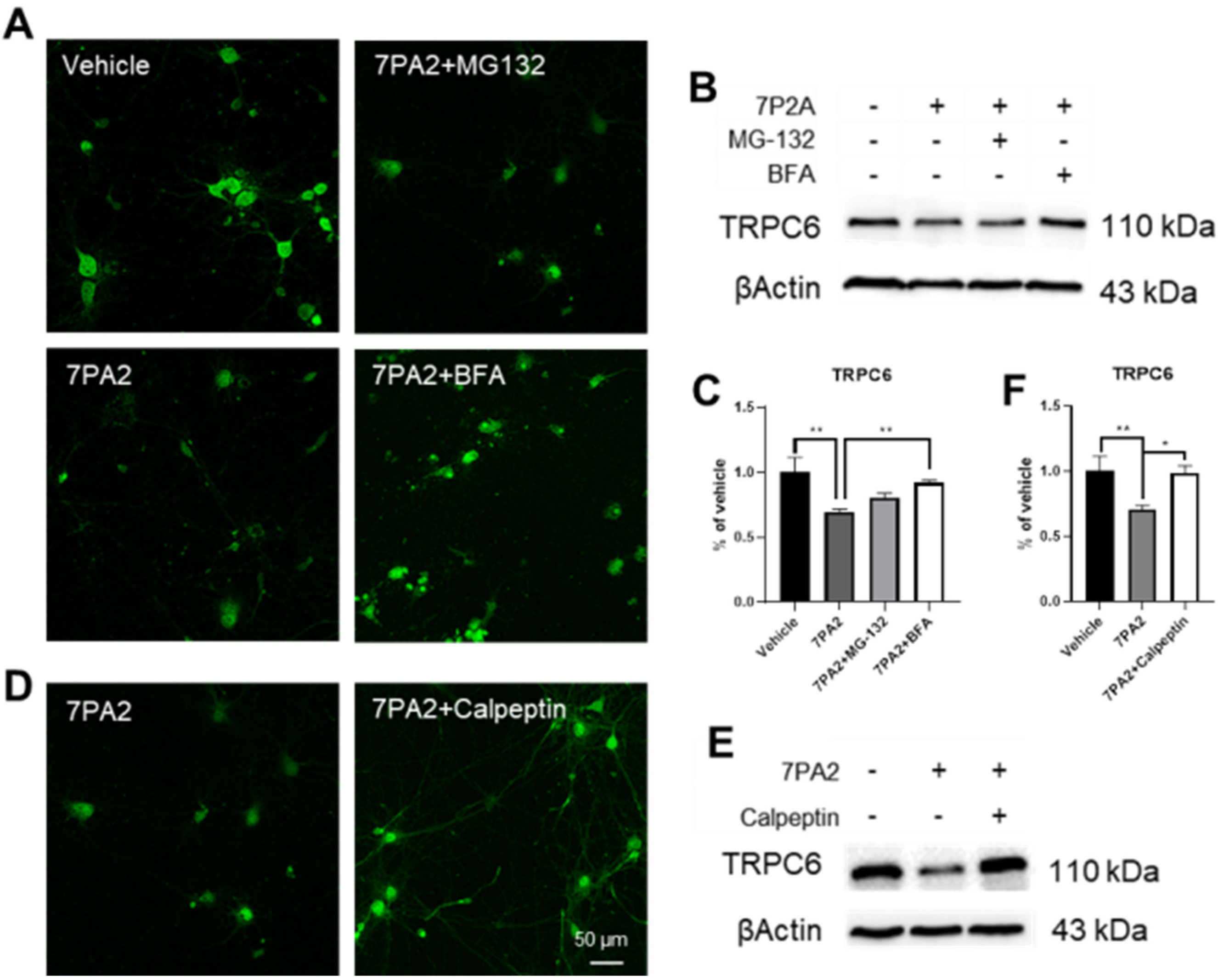

Figure S5. 7PA2 treatment induces TRPC6 protein degradation via the lysosome-endosome pathway. (A, D) Representative images of TRPC6 immunosignals detected at 16 hr after various treatments in primary hippocampal neurons. Neurons were co-treated with BFA (5μg/ml), MG-132 (5 μM), calpeptin (20 μM), and 7PA2. (B, E) Representative immunoblots of TRPC6 protein with lysates prepared from primary cortical neurons at 16 hr after the treatments. (C and F) Quantifications of the WBs from the experiments of panels B and D, based on 3 independent experiments. *p<0.05, and **p<0.01, respectively.

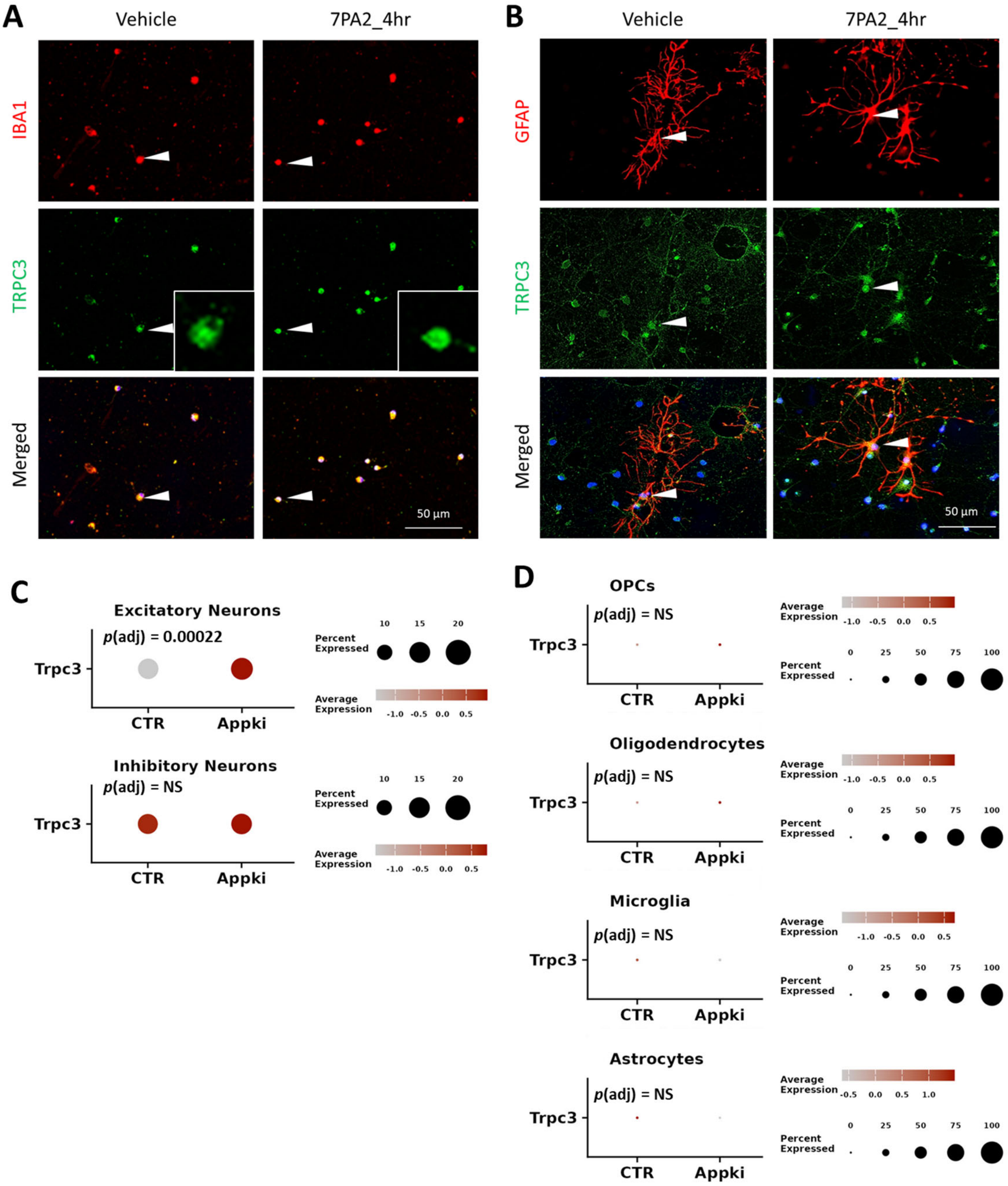

Figure S6. 7PA2 cell treatment increases TRPC3 expression in mature microglia (A) and astrocytes (B): white arrowheads indicate cells with increased TRPC3 immunosignals (Inset in A), and the cell body of astrocytes (right panel B). (C) Selectively up-regulated *Trpc3* gene detected in excitatory neurons by single-nucleus RNAseq analysis. Dot plots showed that the average expression of the Trpc3 gene increased in the excitatory neurons, but not in the inhibitory neurons, of App$^{NL\text{-}G\text{-}F/wt}$ mice compared to control mice. (D) The same snRNAseq data on various cell types. Dot plots show that the average expression of *Trpc3* in oligodendrocyte precursor cells/OPCs, oligodendrocytes, microglia, and astrocytes was not significantly altered in the App$^{NL\text{-}G\text{-}F/wt}$ mice compared to control mice (NS: non-significant). Endothelial cells were not identified in our experimental samples [38].

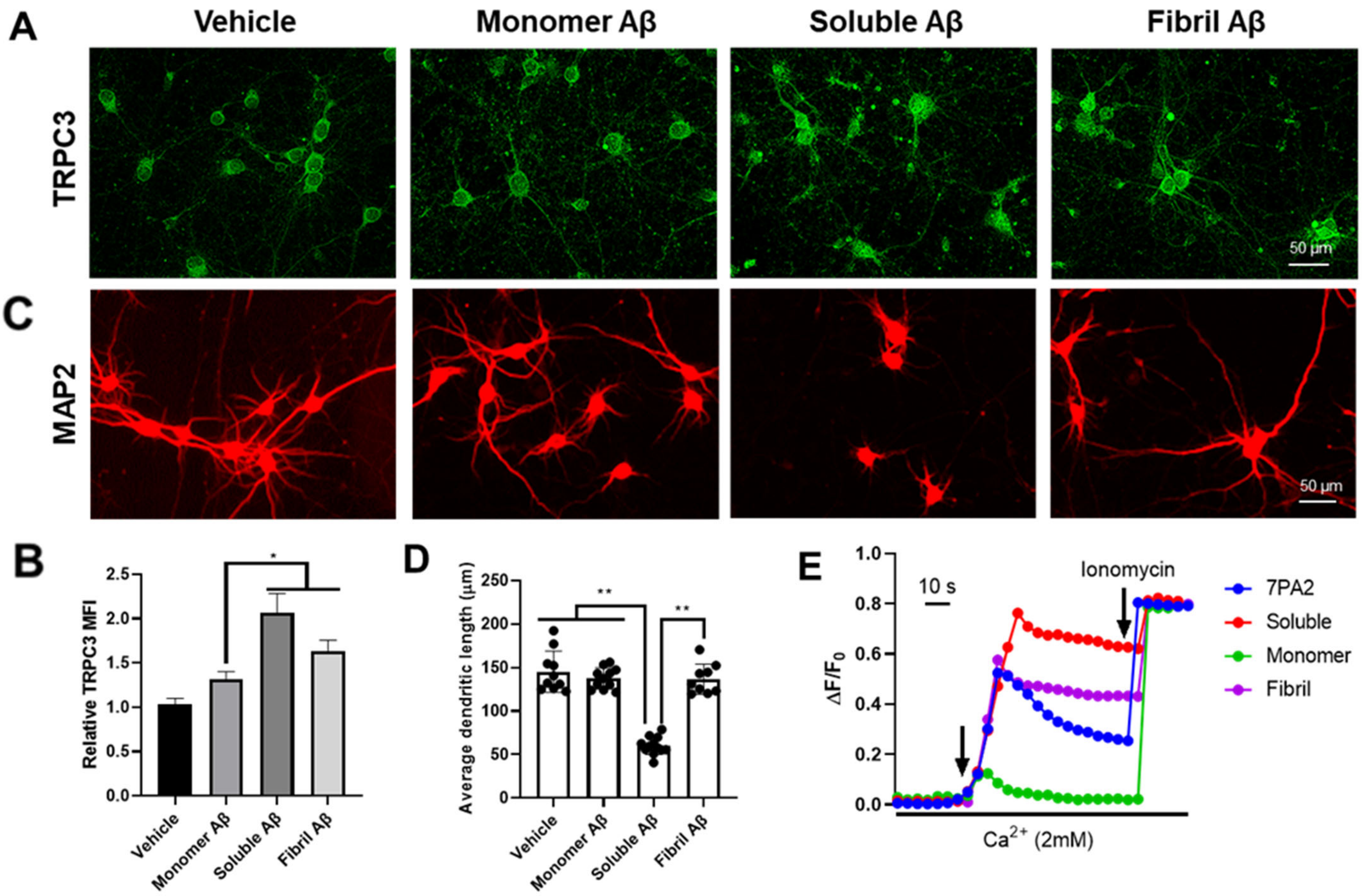

Figure S7. A$\beta_{25\text{-}35}$ oligomers are synaptotoxic to neurons. (A) Representative immunostained TRPC3 images of the hippocampal neurons treated with A$\beta_{25\text{-}35}$ monomers, soluble oligomers and fibrils, all at 10 μM (4 hr) based on the monomeric starting peptide concentrations. (C) Representative immunostained MAP2 images of the treated hippocampal neurons with A$\beta_{25\text{-}35}$ species at 10 μM for 16 hr. (B, D) Quantification of results from experiments in panels A and B, respectively, based on 4 experiments. *P<0.05; **P<0.01. (E) Calcium imaging of the neurons with 7PA2 CM or A$\beta_{25\text{-}35}$ species (10 μM). The left arrow indicates when the 7PA2 or synthetic A$\beta_{25\text{-}35}$ species (10 μM) was perfused, and the right arrow indicates when ionomycin was perfused (2 μM).
